# Epigenetic regulation of serotype expression antagonizes transcriptome dynamics in *Paramecium tetraurelia*

**DOI:** 10.1093/dnares/dsv014

**Published:** 2015-07-31

**Authors:** Miriam Cheaib, Azim Dehghani Amirabad, Karl J. V. Nordström, Marcel H. Schulz, Martin Simon

**Affiliations:** 1 Molecular Cell Dynamics, Centre for Human and Molecular Biology, Saarland University, Saarbrücken 66123, Germany; 2 Cluster of Excellence for Multimodal Computing and Interaction, Saarland University and Max Planck Institute for Informatics, Saarbrücken 66123, Germany; 3 Epigenetics Department, Centre for Human and Molecular Biology, Saarland University, Saarbrücken 66123, Germany

**Keywords:** antigenic variation, epigenetics, ciliate, telomere position effect, heat-shock

## Abstract

Phenotypic variation of a single genotype is achieved by alterations in gene expression patterns. Regulation of such alterations depends on their time scale, where short-time adaptations differ from permanently established gene expression patterns maintained by epigenetic mechanisms. In the ciliate *Paramecium*, serotypes were described for an epigenetically controlled gene expression pattern of an individual multigene family. Paradoxically, individual serotypes can be triggered in *Paramecium* by alternating environments but are then stabilized by epigenetic mechanisms, thus raising the question to which extend their expression follows environmental stimuli. To characterize environmental adaptation in the context of epigenetically controlled serotype expression, we used RNA-seq to characterize transcriptomes of serotype pure cultures. The resulting vegetative transcriptome resource is first analysed for genes involved in the adaptive response to the altered environment. Secondly, we identified groups of genes that do not follow the adaptive response but show co-regulation with the epigenetically controlled serotype system, suggesting that their gene expression pattern becomes manifested by similar mechanisms. In our experimental set-up, serotype expression and the entire group of co-regulated genes were stable among environmental changes and only heat-shock genes altered expression of these gene groups. The data suggest that the maintenance of these gene expression patterns in a lineage represents epigenetically controlled robustness counteracting short-time adaptation processes.

## 1. Introduction

Alterations in gene expression patterns are the regulatory basis for phenotypic plasticity: one single genome may produce quite different phenotypes, thus representing an important mechanism to adapt for alternating environmental conditions including morphological, physiological and behavioural changes.^1^ Similarly, differentiation processes in developmental stages of multicellular organisms allow one single genotype to differentiate into distinct tissues, representing the regulation of gene expression patterns in the meaning of phenotypic plasticity: epigenetic mechanisms have the aim to fix these gene expression patterns rather than to allow subsequent flexibility.^2^

Among the diverse mechanisms regulating alterations in gene expression during phenotypic variation, one can distinguish mechanisms that evolved as a fast reaction to stresses, e.g. the heat-shock response, and adaptations to long-term changes in environments, e.g. such as temperature and food availability. The molecular regulation of the heat-shock proteins (HSPs) represents a tightly controlled transcriptional activation of molecular chaperones that assist proper folding of proteins.^3^ Usually, this transcriptional activation of HSPs occurs in minutes and only for a few minutes, thus being a rapid, programmed and reversible response to stress.

Although it is clear that short-time stress response and long-term phenotypic plasticity are key players for survival during short-time and long-term environmental changes, a general understanding of the complex genetic and epigenetic mechanisms controlling phenotypic plasticity as a response to environmental changes is still missing.^4^ Only few studies addressed the extent of phenotypic plasticity based on genome-wide transcriptomic profiling in response to altered environments.

Adaptation to different environmental temperatures represents one of the most important stress responses of organisms. As ectothermic species may use any environmental heat source to regulate their body temperature thus optimizing their metabolism, some organisms are poikilothermic, meaning that their body temperature is equal to the ambient environmental temperature which challenges the cellular metabolism to work properly in a huge range of temperatures. Depending on the level of temperature variation, adaptation processes have to control survival critical functions like efficient metabolic turnover. In the past, much attention was spend on the composition of membrane lipids during cold adaptation and heat-shocks,^5^ but only few studies investigated transcriptomic changes to identify general metabolic pathways altered during temperature adaptation processes.

Paramecia are unicellular model organisms belonging to the ciliate clade. These are distributed all over the world in all climate areas. However, a single population may have to cope with drastic temperature alterations: on the one hand, these cells are so small that they cannot prevent poikilothermy by increasing body mass/volume ratio, and on the other hand, their natural ecosystem of small ponds undergoes drastic short-time temperature alterations. It is not surprising that paramecia in laboratory cultures can easily be cultivated at a broad temperature range from 4°C to 32°C. Therefore, *Paramecium* represents an excellent model to investigate mechanisms of temperature adaptation, and this ciliate has been subject of several studies investigating heat-shock response of individual genes,^6–8^ but a genome-wide analysis is still missing.

This unicellular model organism has become even more attractive for the characterization of phenotypic plasticity as several epigenetic mechanisms have been described that allow differentiation of lineages into different cell states, which are reminiscent of developmental differentiation processes in multicellular organisms. One prominent example concerns the expression of the mating-type system,^9^ and similarly, but not by the same mechanisms, the antigenic system was described to be epigenetically controlled a long time ago.^10^ Recent studies indicate that the antigenic system is regulated by complex mechanisms involving small RNAs and dynamic chromatin alterations^11,12^ (see Cheaib et al., in preparation): *Paramecium* displays mutually exclusive expression of variable surface antigen genes similar to the phenomenon of antigenic variation in pathogens.^13^ Serotypes, meaning exclusive expression of one antigen gene of the surface antigen gene (SAg) multigene family, are initially triggered by different environmental conditions, e.g. temperature and food availability.^14^ They are stabilized by mechanisms similar to epigenetic differentiation processes, which are under control of an RNAi mechanism likely involved in transcriptional silencing.^11^ It was shown that a telomere position effect (TPE) is required for SAg regulation, suggesting that spreading of telomeric heterochromatin is involved in RNAi-mediated silencing.^12^ Differentiation into distinct serotypes therefore represents an epigenetically controlled manifestation of gene expression patterns, which interestingly can also be passed on to sexual progeny. In contrast to mating-type determination, serotype switches can occur anytime without the need for sexual recombination. Hence, they represent an important example of epigenetically controlled and heritable transcriptomic variations of a single genotype.^13^

To understand the processes of long-term and shocked temperature adaptations in the context of serotype expression, we analysed the transcriptome information of serotype pure cultures to see to which extent the transcriptome becomes altered during temperature adaptation and how this is related to the epigenetically controlled SAg multigene family. We furthermore analysed the subtelomeres of SAg containing chromosomes to characterize a possible heterochromatic spreading from telomeric repeats by transcriptomic analysis.

## 2. Materials and methods

### 2.1. *Paramecium* culture conditions, serotype analysis and heat-shocks

*Paramecium tetraurelia* strain 51 and d4-2 were grown in wheat grass powder (WGP, Pines International Co., Lawrence, KS, USA) infusion medium bacterized with *Klebsiella pneumoniae* supplemented with 0.8 µg/ml β-sitosterol, unless otherwise stated (serotype 51A, 51H of strain 51) and 1 : 1 diluted with Volvic^®^ mineral water (serotype 51D, 51B of strain d4-2). To stabilize/trigger respective serotypes, different cultivation temperatures were chosen: 51A at 31°C, 51B at 24°C/6°C, 51D at 24°C and 51H at 14°C. Serotype expression was verified by immobilization with specific antibodies (1 : 100) (rabbit α-51A/-51B/-51D/-51H) in depression slides. Only serotype-pure cultures (refers to 100% immobilization) were used for further analysis. For heat-shock experiments, serotype-51D cultures were harvested, washed in Volvic^®^ mineral water and exposed to 39°C for 20 min.^8^ 51B cells were selected out of a 51D culture by immobilization with α-51D serum (1 : 100). Individual survived cells were transferred to fresh WGP-medium bacterized with *K. pneumoniae* after washing in Volvic^®^ mineral water. One hundred per cent serotype 51B expressing cultures were cultivated at 24°C. They were then split, and the counterpart was cultivated at 14°C for 1 day before transfer to 6°C.

### 2.2. RNA isolation, cDNA library creation, Illumina sequencing

A total of 150,000 *P. tetraurelia* cells per sample were harvested, washed in Volvic^®^ mineral water and incubated for 20 min at the respective temperature in non-nutrient medium to reduce bacterial occurrence in food vacuoles. Total RNA was isolated using TriReagent^®^ (Sigma-Aldrich, Seelze, Germany). After DNAse I (Invitrogen, Karlsruhe, Germany) digestion and subsequent purification with acid phenol, RNA integrity was verified on an Agilent Bioanalyzer 2100. Poly-A enrichment (NEBNext^®^ Poly(A) mRNA Magnetic Isolation Module) out of 1 µg total RNA and library preparation (NEBNext^®^ Ultra™ Directional RNA Library Prep Kit for Illumina) were carried out according to manufacturer's recommendation using 11 PCR cycles of library enrichment. Multiplexed libraries were 100 nt paired-end sequenced with an average of 14 Mio reads per sample on Illumina HiSeq 2500 platform. Reads were demultiplexed with bcl2fastq (v1.8.4) and trimmed for adaptor contamination and low-quality bases with the cutadapt (v1.4.1)^15^ wrapper trim_galore (v0.3.3). All raw data were deposited at the European Nucleotide Archive (ENA, http://www.ebi.ac.uk/ena) under study accession no. PRJEB9464.

### 2.3. Annotation of HSP70 isoforms and phylogenetic reconstruction

HSP70 candidates were identified by a proteome-wide search against the Pfam library of hidden Markov models.^16^ Hits were extracted for PF00012 (HSP70) using an E-value threshold of 3.6E−23. Amino acid sequences were aligned with ClustalW, and neighbour-joining trees were calculated with MEGA6^17^ with 1,000 bootstrap replicates, based on the Poisson model and after complete deletion of gaps.

### 2.4. RNA-seq expression analysis

We quantified gene expression levels using the Sailfish algorithm.^18^ Briefly, Sailfish uses transcript sequences to build a kmer index over all annotated transcript sequences. RNA-seq reads are decomposed into kmers and projected onto kmers of transcripts; then expression levels of all transcripts are deconvoluted. Finally, transcript expression levels of the same gene are summarized to give a gene expression estimate. As a normalized expression metric for plots, we used Reads per Kilobase per Million mapped reads (RPKM) reported by Saillfish. We used Sailfish version 0.6.2 with the following command lines:

sailfish index -t <ref_transcripts> -o <out_dir> -k 20 for building transcriptome index file and

sailfish quant -i indexfile -l ‘T = PE : O =><:S = AS’ -1 sample_R1.fastq -2 R2.fastq -o Sample1 for strand-specific quantification of read files. We downloaded the *P. tetraurelia* cDNA file from the Ensembl protists database release 26 (assembly version GCA_000165425). The quantification was done for 39,635 genes.

After gene expression quantification, differential gene expression analysis was conducted using DESeq2 version 3^19^ in R. Following the DESeq2 guidelines, absolute read counts per gene as obtained from Sailfish were normalized between samples to account for differences in sequencing depth. Linear models to assess DE status were fit for each gene with DESeq2 using a negative binomial distribution. After multiple testing correction,^20^ genes with false discovery rate (FDR) <0.01 were considered as differentially expressed.

### 2.5. Transcriptome expression landscape visualization and analysis

To visualize and compare the transcriptome expression landscape for the different conditions, we applied a self-organizing map (SOM) approach using the oposSOM package.^21^ An SOM is a machine learning technique for dimensionality reduction, which transfers a high-dimensional data matrix (expression of all genes across different samples) to a lower dimension.

The oposSOM package decomposes the data into clusters of correlated sets of genes, called metagenes, which are arranged in a fix order (here we use a lattice with dimension 20 by 20). The expression of a metagene represents the average expression of all genes in the cluster. Metagene expression values in the individual samples provide mosaic pictures visualizing condition specific over- and under-expression in terms of characteristic colour-coded textures, which allows the direct comparison of the expression of individual samples in a simple and intuitive way.

TPM scale expression levels were log-transformed as pre-processing step for training the SOM. After training, we had sample-specific transcriptome expression landscapes as well over- and under-expressed hot spots for every sample. Sample-specific transcriptome landscapes represent the expression level of the metagenes at the respective sample while preserving the topological order of the metagenes in high dimension. Metagenes are grouped into co-regulated hot spots of similar up- or down-regulation using the following criteria: if Δ*e* > MaxEXP * 0.9, where Δ*e* is the expression level of the metagene under consideration and MaxEXP denotes the maximum expression of all metagenes, then the metagene is classified as over expressed. Analogously if Δ*e* < MinEXP * 0.9, where MinEXP denotes the minimal expression of all metagenes, the metagene is classified as under-expressed. Metagenes with the same classification that group together in the SOM are called a regulated spot.

Hierarchical clustering (average linkage mode) of expression values was conducted using R, heatmap2 function. We used two different setups for the creation. The first was based on Euclidean distance computation, which measures the distance between all entries in the expression vectors. The second was based on 1-Pearson correlation, which measures if genes follow similar trends independent of their differences in expression values. In all cases, no scaling was used.

### 2.6. Gene ontology enrichment

The gene ontology (GO) term association file has been created by processing the parameciumDB.gff3 file, downloaded from the *Paramecium*DB database.^22^ Differentially expressed genes (DEGs) for every pairwise condition were divided into up- and down-regulated genes. We conducted GO enrichment analysis using Ontologizer (version 2.1) software using the Parent-Child-Union method for each set of up- or down-regulated genes in each pairwise comparison.^23^

## 3. Results and discussion

### 3.1. Experimental design

It was the aim of this study to characterize gene expression patterns following epigenetically stabilized serotype expression in *P. tetraurelia*. We therefore analysed the transcriptomes of four different serotype pure cultures (51A, 51B, 51D, 51H) and furthermore characterized transcriptome alteration during cold adaptation, starvation and heat-shocks to compare epigenetically controlled gene expression patterns to short-time adaptation processes.

All experiments were started with serotypically pure cultures, meaning that 100% of cells expressed one single surface antigen obviously by 100% immobilization to a specific antiserum.

**Figure 1.**
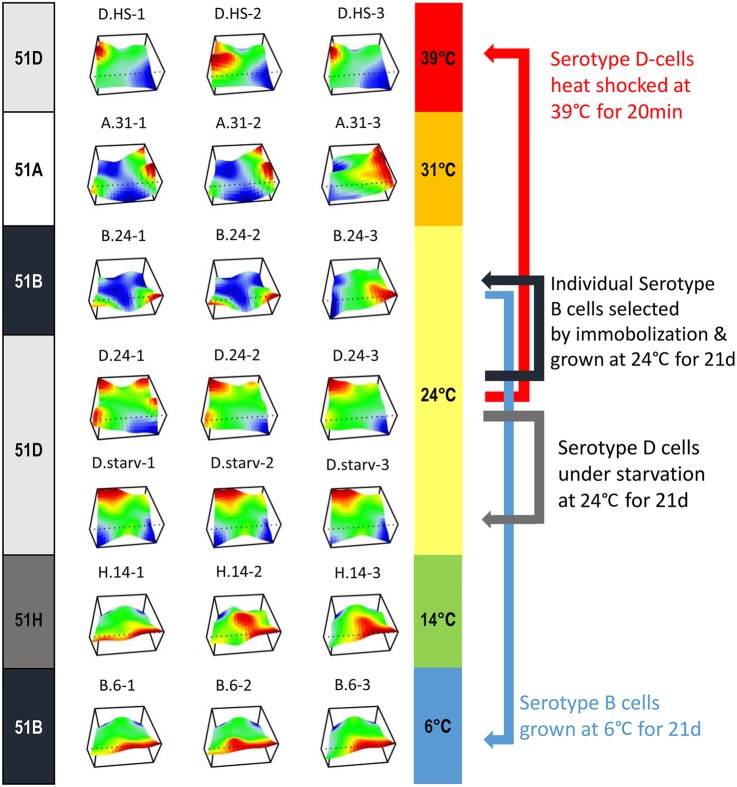
The experimental design and transcriptional landscape of individual cultures. The transcriptional landscape of analysed cultures is illustrated as a 3D heatmap of grouped genes. On the left side, the actual serotype is shown, meaning the surface antigen to which antiserum 100% of the cultures showed positive immobilization reaction at the time of RNA isolation. On the right side, the individual cultivation temperature is indicated (except the heat-shock cultures that were cultured at 24°C and were transferred to 39°C only for 20 min).

In this study, we analysed seven distinct conditions, three biological replicates each (Fig. 1):

- Serotype 51A expressing cultures cultivated at 31°C for >6 months (A.31).
- Serotype 51D expressing cultures cultivated at 24°C for >6 months (D.24).
- Serotype 51H expressing cultures cultivated at 14°C for >6 months (H.14).
- Three aliquots of the D.24 cultures subjected to a 20 min heat-shock at 39°C (D.HS).
- Three cultures (B.24) were grown from 51B expressing individuals, which were selected from the D.24 cultures by immobilization with anti-51D serum. Surviving individuals were subjected to autogamy and further cultivated at 24°C for 3 weeks.
- Three aliquots of the 51B cultures that were gradually adapted for lower temperatures (starting at 24°C, slow cool down to 14°C within 24 h, slow cool down to 6°C in 24 h) and then cultivated 3 weeks with permanent food supply at 6°C (B.6).
- Three aliquots of the D.24 cultures were subjected to autogamy and subsequently starved at 24°C for 3 weeks (D.starv).

Replicate experiments are denoted with numeric suffixes, for example, the three replicate cultures of B.6 are denoted B.6-1, B.6-2 and B.6-3. As serotypes are expressed throughout the life cycle and usually in most individuals of a population, all cultures analysed in this study represent vegetatively growing non-synchronous cultures, thus representing individuals of different cellular age.

To ensure that transcriptome differences do not result from activation of genes specifically activated during sexual recombination (autogamy), all cultures were checked for fragmented nuclei by DAPI staining prior to RNA isolation. In addition, Supplementary Fig. S1 shows gene expression profiles of different (early, intermediate, late induced) autogamy-related genes showing no significant activation in any culture.

### 3.2. Analysis of DEGs

As *P. tetraurelia* had at least three whole-genome duplications, there are a number of genes that appear in more than one highly similar copy in the genome.^24^ This complicates gene expression estimates as reads may map to more than one location in the genome, potentially biasing estimates of non-expressed genomic copies of a gene. We used the Sailfish algorithm,^18^ which uses a global approach to optimize read placements on genes, to avoid false-positive expression signals of silent genes. We did a qualitative investigation by looking at the two highly similar genes SAg 51D and 51J (92.2% sequence identity) that we had previously measured with qPCR.^12^ Whereas the 51D gene has the highest expression in D serotype cultures, the 51J gene is silent, despite the highly similar sequence, and the same pattern is observed for the Sailfish estimates and with qPCR.

After quantifying all gene expression values in the samples, we used a dimensionality reduction approach with SOMs to compare the expression landscapes between different conditions and their replicates (see Section 2). Figure 1 shows the transcriptional landscapes of all analysed cultures in 3D heatmaps. As expected, individual replicates are highly similar over the complete landscape, but may differ for smaller, individual gene groups, as seen for cultures A.31-3 and B.24-3. We decided to proceed the subsequent analysis also with these samples rather than to omit them as they represent biological fluctuations that cannot be neglected, but we are aware that some DEGs may become masked in the subsequent analyses merging all three replicates.

At first glance, a similar but not identical landscape can be observed in the maps of the two cold-adapted experiments cultured at 6°C and 14°C (B.6 and H.14). More surprisingly, the serotype pure B.24 and D.24 cultures show entirely different patterns although cultivated under identical environmental conditions. In contrast, the heat-shocked and the starved cultures still show similarity to their origin, the D.24 samples. To describe the relationship between the individual cultures and conditions, we analysed DEGs in more detail.

**Figure 2.**
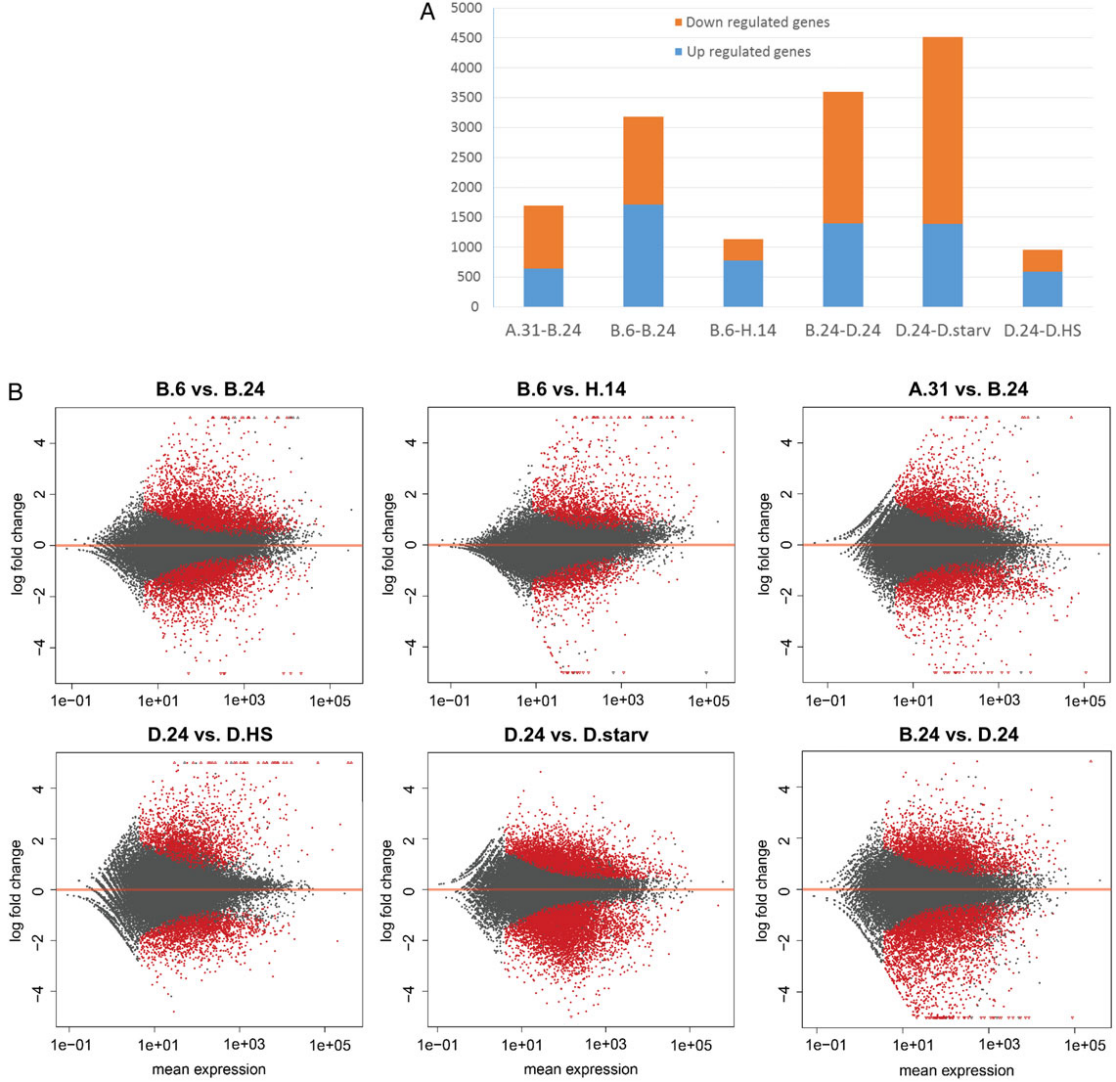
MA plots of mean differential gene expression. (A) The number of DEGs between individual comparisons (FDR ≤ 0.01). (B) MA plots of fold change expression level (*y*-axis) against expression level (*x*-axis). Each point represents a transcript; those with significant differential expression (FDR ≤ 0.01) are indicated in red. The cultures compared are indicated above each plot. A summary of all possible comparison between cultures can be found in the Supplementary Fig. 2.

Using estimated gene read counts from the Sailfish analysis, we used the DESeq2 algorithm to compute pairwise DEGs between different conditions (see Section 2) and set a cut-off for DEGs at an FDR value of 0.01. Figure 2 shows complete numbers (Fig. 2A) and MA plots (Fig. 2B) of interesting comparisons as a detailed analysis of fold changes in relation to mean expression levels (further comparisons between conditions can be found in Supplementary Fig. S2). The plots support observed differences between the B.24 and D.24 cultures with 2,202 genes being significantly down-regulated in serotype 51D cells. As single 51B cells spontaneously appeared in the 51D cultures and were selected by immobilization, these differences suggest that the altered SAg, specific for the serotype, is not the only DEG but many more genes show altered gene expression. In contrast, the other two sets of variants of the D.24 condition, heat-shock and starvation, show less differences: in the former 587 up-regulated genes total in the latter case of starvation 3,123 genes; however, most of them showing a moderate down-regulation of ∼log-fold change −2. The cold-adapted transcriptome of the B.6 cultures shows 1,709 down- and 1,472 up-regulated genes and therefore obviously a higher degree of alteration during cold adaptation compared with starvation. However, there is still a difference between the two sets of cold cultures B.6 and the H.14, which were adapted for a much longer time. In addition, the MA plot comparing A.31 and B.24 shows many genes that are differentially regulated although the total number of significantly DEGs is lower (639 up and 1,058 down) compared with the B.24 and D.24 comparison.

### 3.3. Comparison of individual transcriptome similarity

**Figure 3.**
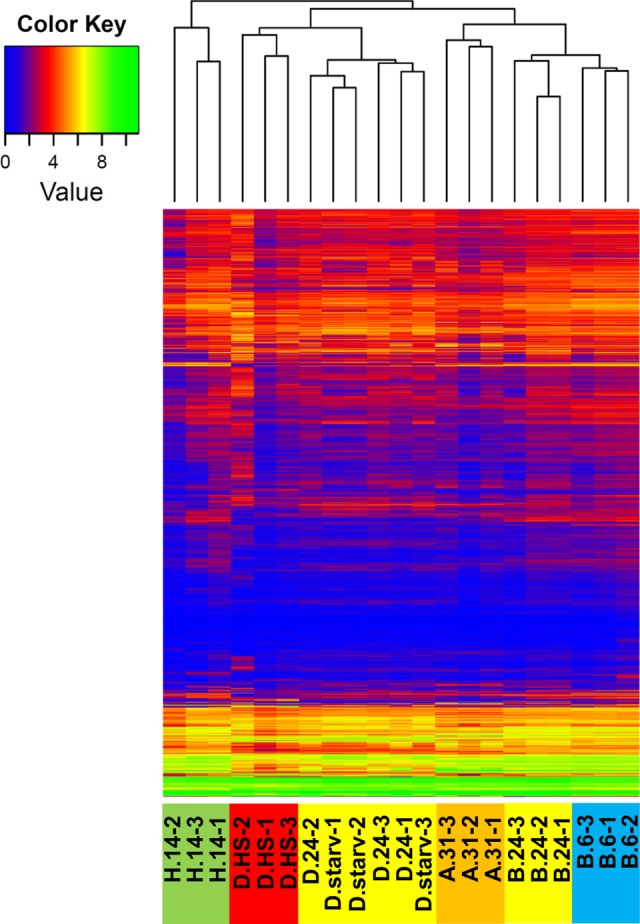
Relationship between transcriptomes of cultures and their replicates. Clustering heatmap showing expression levels (colour key on the left: green high and blue low expression) of the entire transcriptome per sample (bottom label). The dendrogram (top) indicates the global similarity of the transcriptome profiles using hierarchical clustering (Euclidean distance, average linkage) on the log-transformed expression values.

To get an overview of the global similarity between the different transcriptomes, we used hierarchical clustering (Euclidean distance, average linkage) on the log-transformed TPM estimates. Figure 3 shows the clustering result as a heatmap, indicating transcription levels of all genes clustered according to their common expression behaviour in all analysed libraries. The dendrogram above the heatmap reflects the overall similarity between the transcriptome profiles. Excluding the D.24 and D.starved cultures, this analysis clearly clusters the individual biological replicates although few replicates (e.g. H.14-2 or D.HS-2) show divergence as indicated by comparing transcriptomic landscapes in Fig. 1. Comparing the D.24 and D.starv cultures, our analysis indicates that these still have similar transcriptomes: the D.starv cultures have been separated from the well-fed D.24 cultures and maintained without feeding bacteria for 3 weeks (Fig. 1).

As a first insight in the general comparison of the transcriptomes, the indication from the MA plots can be confirmed, because the two serotypes B.24 and D.24 that were cultured both under identical environmental conditions show huge differences and are clearly separated in the dendrogram (Fig. 3). This suggests indeed that the differences observed here are not due to the environmental temperature or other conditions, e.g. food or composition of medium, which were identical for both. In support of the latter conclusion, the two cold-adapted cultures B.6 and H.14 are clearly separated in Fig. 3 as the overall similarity of the transcriptome reveals large differences and clusters the B.6 cultures close to the B.24 conditions, from which they were derived.

At first glance, the separation of the B.6 and H.14 transcriptomes in the dendrogram appears to be in conflict with the fact that both share the lowest number of DEGs (Fig. 2A). In comparison to the distance-based clustering in Fig. 3, Supplementary Fig. S3 shows a clustering obtained using Pearson correlation of gene expression values (see Section 2.5). Using correlation allows comparing gene expression patterns, despite their absolute differences in expression. In agreement with the low number of DEGs in the B.6/H14 comparison, both transcriptomes are clustered together in the second cluster analysis. We therefore conclude that a common mechanism of cold adaptation occurred in both the H.14 and the B.6 cultures (indicated by the low number of DEGs and clustering by Pearson correlation), but that expression of many genes in the B.6 cultures remains similar to expression in the parental B.24 cultures (see further analysis of these genes below).

The lowest number of DEGs can be seen between D.24 and its heat-shocked portions D.HS. As the MA plot in Fig. 2 shows, the heat-shock is characterized mainly by strong activation of a certain number of genes, which explains the separation of the D.HS transcriptomes from the two other conditions of the serotype D cultures at 24°C in the dendrogram (Fig. 3).

Figure 3 apparently shows that all cultures undergoing an environmental change (B.6, D.starv and D.HS) still cluster to their originating cultures. Although we did not necessarily expect a very high number of DEGs in the heat-shocked cultures, one would have expected greater differences in starvation and especially in the B.6 cultures. This suggests that these changes in the 3-week period led only to a partial rearrangement of the previously governing gene expression patterns.

### 3.4. Cold adaptation by alterations of metabolic, DNA metabolic and translational alterations

**Figure 4.**
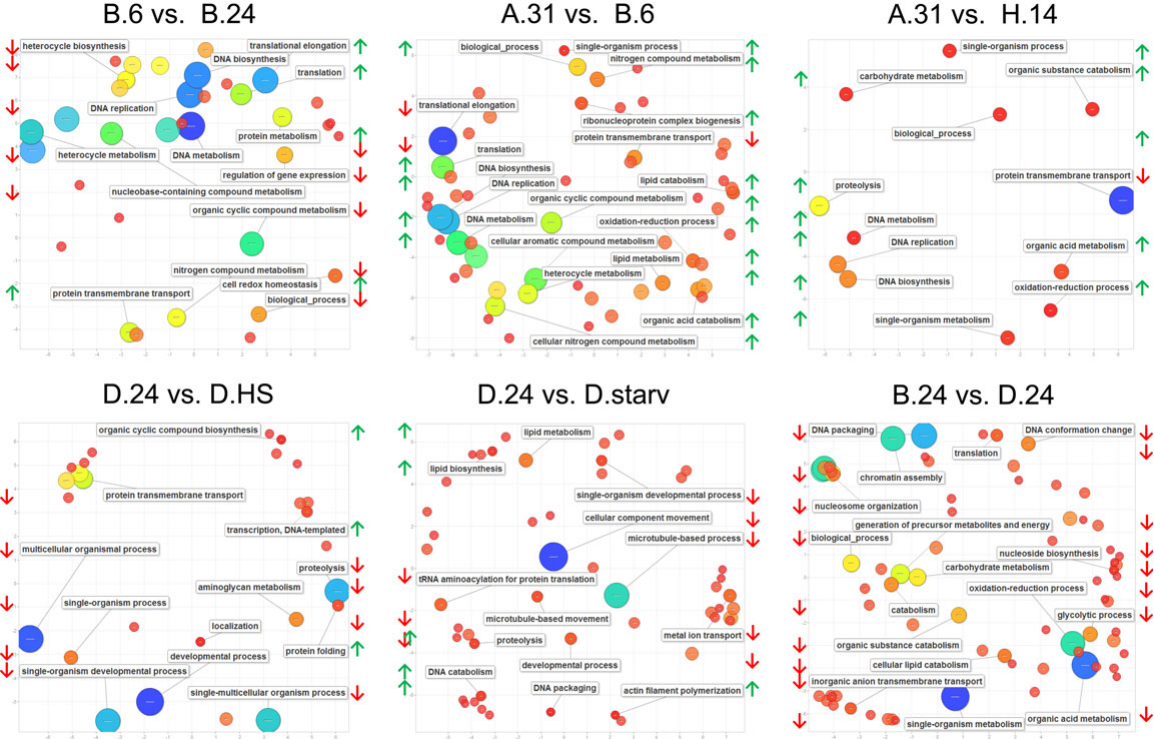
GO enrichment analysis results. Principal component analysis (PCA) scatter plots of GO representatives of biological processes generated by the REVIGO tool that summarizes the list of differential expressed GO terms (FDR ≤ 0.02) by removing redundant terms.^25^ The distance between circles (representing individual GO terms) indicates the relationship between terms: closer distance means closer relationship. Bubble colour indicates significance of differential expression of an individual GO term (red low and blue high); the size (in log_10_ *P*-value) indicates the percentage of genes annotated with a term in the reference database (UniProt) and thus indicates more general terms (large) and more specific ones (small). Most significant terms are labelled in the individual plot as well as representatives for groups of terms with lower significance. Green and red lateral arrows indicate the direction of regulation (up or down).

Figure 4 shows enrichment of GO terms for biological processes and individual comparisons (complete lists of differential GOs for these comparisons can be found in Supplementary File 5).

In all three warm/cold comparisons made here (B.6 vs. B.24; A.31 vs. B.6; A.31 vs. H.14), DNA metabolic GO terms including DNA biosynthesis and replication (initiation) are strongly up-regulated in the respective cold cultures. In addition, also oxidation/reduction processes are up-regulated in all cold-adapted cultures (only in the B.6 vs. B.24 comparison slightly below our set threshold). As this indicates higher energy metabolism, maybe due to the cold temperatures, this goes along with increased lipid catabolism in the B.6 cultures and with increased carbohydrate catabolism in H.14 cultures. As the representation of GO terms in Fig. 4 often reduced individual processes to parental GO terms (e.g. biological process), this indicates indeed that temperature adaptation alters many different processes, which require further investigation by subsequent experiments.

### 3.5. GO term enrichment in starvation, heat-shock and serotype comparison

The GO analysis indicates that starvation most significantly triggers microtubule-based processes and movement of cellular components, thus suggesting rearrangements of the cytoskeleton, probably because these starved cells do not undergo cell divisions. Up-regulated processes are DNA catabolism and lipid metabolism, indicating increased usage of lipids for energy metabolism.

In the heat-shocked cultures, the most significant up-regulated GO term is ‘protein folding’ (GO: 0006457, Supplementary File 5), which involves the heat-shock response chaperones in agreement with our expectations. The GO enrichment displayed in Fig. 4 clearly shows that many other global processes are down-regulated. In the special situation of the heat-shock, these down-regulations may not be necessarily considered as controlled regulation of gene expression but also a by-product of thermal instability of individual mRNA species as discussed later.

In general, the B.24 and D.24 comparison shows that many processes are down-regulated: the most significant one is again ‘oxidoreductase activity’ (GO: 0016491), indicating that the D.24 cultures have a lower energy metabolism compared with the B.24 cells, despite identical environmental cultivation conditions and thus food supply. In this comparison, also GO terms concerning ‘chromatin assembly’ become apparent in Fig. 4. A closer look reveals that chromatin-modifying enzymes are differentially expressed, namely genes of three histone deacetylase isoforms, chromodomain containing proteins and nuclear assembly proteins, and surprisingly the core histones themselves (Supplementary Fig. S4A). Several isoforms of histone genes H2A, H2B, H3 and H4 are down-regulated in the D.24 cultures. Supplementary Fig. S4B and C show, for histone H3, that only those isoforms are down-regulated that are constitutively expressed throughout the life cycle and that the expression of developmental specific isoforms is not affected.

Further wet lab work has to clarify whether the global down-regulation of the core histones in our RNA-seq data indeed indicates a genome-wide loss of nucleosome occupancy: histone gene expression was described to be complex and to occur at several levels including post-transcriptional regulation.^26^ Therefore, our data do not allow for conclusions that indeed histone protein levels are strongly regulated here. However, if lower nucleosome occupancy would be true in the D.24 cultures, the high number of DEGs to the B.24 cultures might also be the result of a complex re-organization of the macronuclear chromatin.

In yeast, cellular ageing was described to be accompanied by a loss of nucleosomes and a global transcriptional up-regulation,^27^ which is different to our observations as the D.24 cultures show more down-regulated genes. As the cells of the D.24 cultures were not synchronized in cellular age, we cannot rule out an age-dependent regulation of histones, although the down-regulation is maintained in the D.starv cultures, which represent 10–20 division old F1 individuals of the D.24 cultures.

A similar result of decreased expression of the core histones was recently observed in *P. tetraurelia* by microarray analysis during silencing of an RNA-dependent RNA polymerase 3 (RDR3), which resulted in a co-expression of SAgs accompanied with decreased expression of the core histones^12^ (M. Simon, unpublished data). As these cultures were of the same cellular age as the controls, any age-dependent influence can be ruled out in these experiments. Surprisingly, we observe a similar phenomenon of low core histone expression here in wild-type cultures of different serotypes. As *RDR3* silencing is also accompanied with drastically decreased cells divisions,^11^ this was also observed for D.24 cultures, which showed slightly fewer cell divisions per day compared with the B.24 cultures (data not shown). As reduced histone supply was described to extend the S phase in *Drosophila*, the delivery with core histones turns out to be a crucial component regulating the cell cycle.^28^ Although the ciliate macronucleus divides amitotically, the prolonged cell cycle fits to our observations in the D.24 cultures and *RDR3*-silencing cultures.

In support of this conclusion, the heat-shocked derivatives of the D.24 samples show a down-regulation of the core histones (Supplementary Fig. S4). Although we did not analyse the division rate of the heat-shocked cells, the down-regulation of the histones is consistent with a recent characterization of the heat-shock in the unicellular algae *Chlamydomonas* showing decreased histone expression by proteomic analysis; in this particular case, cells show an immediate stop of cell divisions.^29^

It seems tempting to speculate here that *Paramecium* controls cell division also by histone supply although the underlying regulatory mechanisms and the involvement remain unclear, especially the involvement of the *RDR3-*associated RNAi machinery. In this context, recent work in the ciliate *Stylonychia* demonstrated that the knock down of the *Stylonychia* Piwi resulted in down-regulation of individual H3 isoforms, thus providing another example for the connection of RNAi and histone expression.^30^ Further studies have to clarify the role of histone gene expression and RNAi components as this would represent a powerful and new parameter of epigenetic influence to chromatin and genome integrity.

### 3.6. Constitutive and regulated expression of HSP70 isoforms

**Figure 5.**
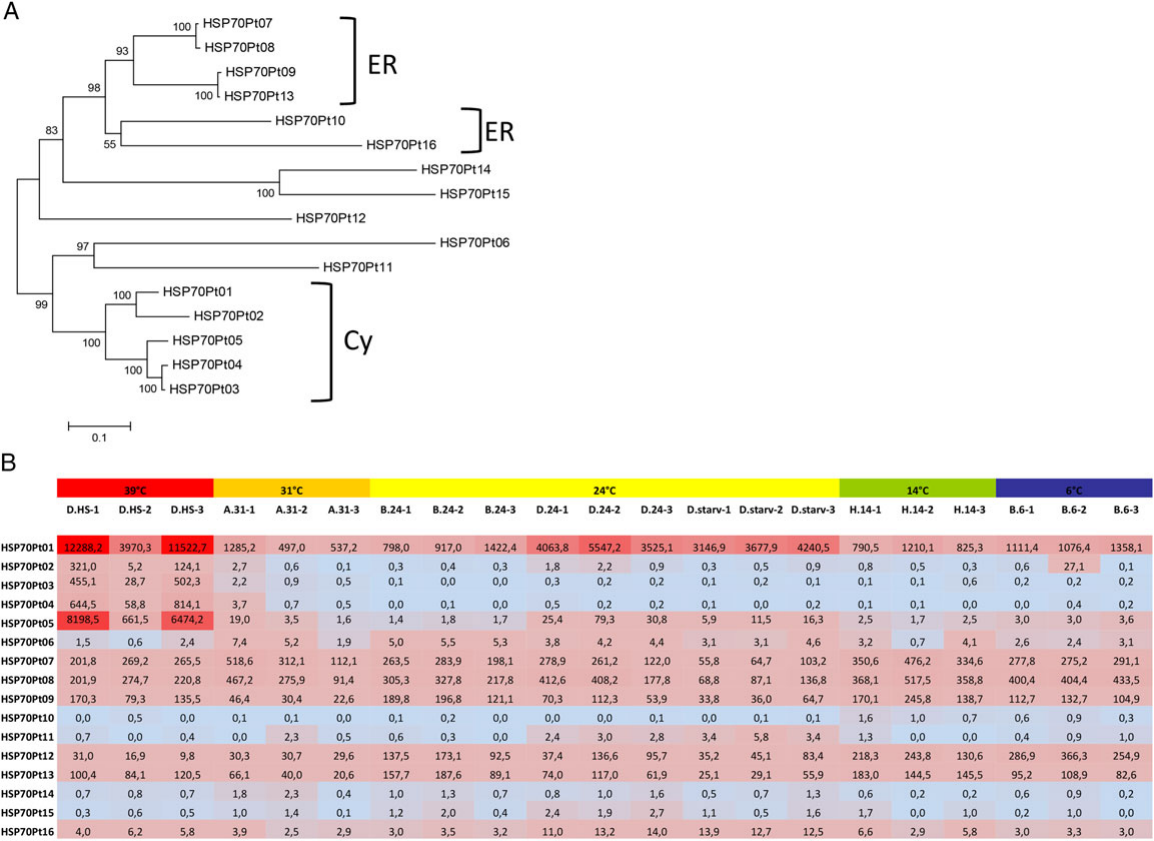
Expression level of heat-shock (HSP70) genes. (A) Neighbour-joining tree of HSP70 proteins of *Paramecium tetraurelia* (with 1,000 bootstrap replicates). The tree includes 10 previously described HSP70 genes (HSP70Pt01-10)^6,7^ and additionally identified HSP70 isoforms (HSP70Pt11-16). Putative localization of isoforms is based on similarity analysis to HSPs in other organisms and on the classification in Krenek et al.^7^ ER-endoplasmic reticulum, CY-cytosol. (B) TPM normalized expression values for all described HSP70 isoforms in all samples are shown, and cells are coloured according to high (red) or low (blue) expression.

Ten HSP70 isoforms have been described earlier for *P. tetraurelia,*^6,7^ and we added six more to the annotation, which were identified by a proteome-wide Pfam search (see Section 2 for details). Figure 5A shows the phylogenetic relationship between these isoforms and their putative subcellular localization. Figure 5B illustrates the TPM normalized expression levels of these HSP70 isoforms, where heat-inducible up-regulation can only be observed in the cytosolic group, in agreement with a previous analysis.^7^ All other HSP70 isoforms show constitutive expression.

The only cytosolic HSP70 isoform that shows high expression in all cultures is HSP70Pt-01, and interestingly this isoform also shows a second expression peak in D.24 and D.starv cultures. This does not seem to be due to the cultivation temperature, as lower expression can be observed at the same cultivation temperature in B.24 cultures (Fig. 5B). Similarly, also HSP70Pt-05 shows this activation in D.24 and D.starv cultures, however to a lower degree.

Interestingly, a continuous up-regulation of HSP70 isoforms was recently also reported in *Paramecium* in response to endosymbionts, another form of external stress to the cell. The transcriptome comparison of *Paramecium bursaria* without and with endosymbiotic *Chlorella* ssp. algae revealed individual HSP70 isoforms up-regulated,^31^ and similar findings were also reported for infections with bacterial endosymbionts in *Paramecium caudatum*.^32^ Other studies suggest that bacterial symbiont bearing paramecia show an increased stress resistance,^33^ and the authors concluded that permanent HSP70 activation awards increased stress tolerance. Interestingly, symbiont-induced expression of HSP70 isoforms is shown to be permanent even after the removal of bacteria by antibiotics, indicating an epigenetic manifestation of this acquired gene expression.^34^ Here, our results support a permanent activation of an individual HSP70 isoform (HSP70Pt-01) in D.24 as well as the D.starv cultures but not in other samples.

### 3.7. Expression pattern of the surface antigen multigene family

**Figure 6.**
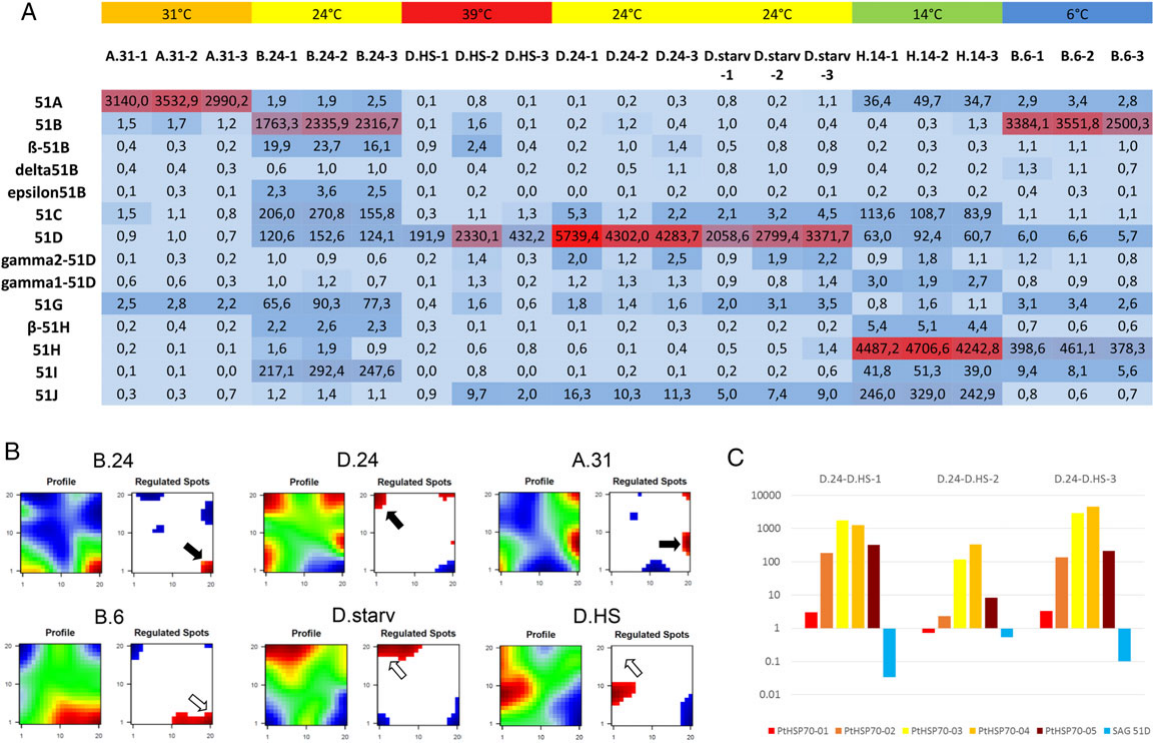
Expression level of SAgs. (A) TPM normalized expression values for members of the SAg multigene family are shown in the table, and colour shading indicates relative expression level (dark blue—low, red—high). (B) Identification of regulated spots (gene clusters showing co-regulation in all analysed samples) in serotype pure cultures. Plots on the left show the global profile, plots on the right show regulated spots. (blue—low expression, red—high expression). The samples are indicated above the plots, a representative replicate is shown. Black arrows indicate the regulated cluster containing exclusively the actual expressed SAg (SAg 51B in B.24; SAg 51D in D.24; SAg 51A in A.31). The open arrows in the second row (B.6, D.starv, D.HS) indicate the position of the regulated spot of the cultures origin (B.24; D.24 and D.24). (C) Fold change expression level of cytosolic HSP70 isoforms and the 51D surface antigen gene of the individual three replicates of the heat-shocked cultures.

As mentioned above, serotypes represent a special kind of differentiation process manifesting a gene expression pattern of the SAg family by epigenetic mechanisms: only one gene is expressed at a time.^35^ The most dominant environmental factor triggering serotype expression was described to be the temperature, and once induced they can be transferred to different cultivation conditions without serotype switches, thus indicating a self-stabilizing gene expression mechanism. Recent studies demonstrated that the core family consists of eight genes, some of them with a certain number of isoforms, indicated by Greek letters.^12^ In all our experiments we started with serotype pure cultures, meaning 100% of the cells responded to specific antiserum in the immobilization reaction. Figure 6A shows the gene expression levels based on TPM normalization. The figure shows high expression levels of the individual SAg that corresponds to the antiserum with which the cells reacted. This is not surprising for the A.31, B.24, D.24 and H.14 cultures. However, the B.6 cultures, which represent an aliquot of the B.24 cultures, still retain high expression of the 51B gene. This means that the expression of the 51B gene shows stable expression, even after 3 weeks at a temperature of 6°C. Typically, *P. tetraurelia* stock 51 expresses the 51H gene at low temperatures.^36^ Our data show that the 51H gene indeed has increased expression in the B.6 cultures, nevertheless the isolated cells still responded 100% to the 51B antiserum. This means that the actual serotype persists even drastically temperature changes. From our experience, it is likely that some individuals of the B.6 cells will indeed shift to 51H after several weeks more of cultivation at 14°C or 6°C, but production of serotype pure cultures will require the selection of 51H expressing individuals. In such a case, serotype shifts can indeed be triggered by temperature alterations, but the selection of individual transformants is required.

Comparing the B.24 and B.6 transcriptomes in the heatmap representing the entire transcriptome, Fig. 3 indicates that many genes maintain their expression pattern, thus explaining the general high similarity. Additionally, the D.24 cultures are still highly similar to the D.starv cultures, and the serotype expression here indicates permanent expression of the 51D gene although slightly reduced (Fig. 6A). For these two examples of environmental alterations (food limitation: D.24 vs. D.starv) and extreme temperature shift (B.24 vs. B.6), our data show that the transcriptomes are indeed much more similar compared with the selected individuals of spontaneous transformants, namely the selected 51B expressing cells from the D.24 cultures that were cultivated under identical conditions.

This suggests that spontaneously occurring transcriptome alterations, which may be regulated by epigenetic mechanisms similar to the serotype system, alter gene expression in *Paramecium* to a much higher degree compared with short-time adaptation to environmental changes.

### 3.8. Many genes show co-regulation with the dominant SAg and sensitivity to heat-shocks

To see whether other genes are co-regulated with the surface antigens, we defined ‘regulated spots’ in the transcriptomes, which are characteristic sets of lowly or highly expressed genes per condition (Fig. 6B). Genes that are part of a spot show not only coherent up- or down-regulation in one individual sample, but moreover they are forming a group of co-regulated genes that behave similarly among all analysed conditions and samples.

These data show that a huge number of genes are indeed co-regulated with individual antigens. These groups contain a large number of genes: on average between the replicates 2,468 genes in A.31, 1,180 in B.24 and 1,860 in D.24, thus representing a high percentage of the genome. In case of the B.24/D.24 comparison, the visualization of the regulated spots almost shows a mirror image of one another, thus explaining the clear separation of both states in the dendrogram (Fig. 3); it is still surprising that this difference is not due to environmental alterations but more likely due to spontaneous alterations. The short-time derivatives of these cultures (B.6 and D.starv) still show these spots (open arrow), although the pattern seems slightly different (Fig. 6B), thus indicating that a huge number of genes follows the expression behaviour of the SAg family and shows stability to environmental alterations within 3 weeks.

Surprisingly, only the heat-shocked cultures (D.HS) alter serotype expression and, moreover, show a loss of the highly expressed spot of regulated genes, which was characteristic for all 51D expressing cultures. This means that a heat-shock of 20 min alters the expression of genes, which we discussed to be co-regulated with the epigenetically controlled SAg family.

Comparing the transcript level of the cytosolic HSP70 isoforms per replicate to the expression levels of SAgs in Fig. 6C, the 51D mRNA is drastically reduced after 20 min heat-shock. This is apparent in the D.HS-1 and -3 cultures, and only to a lesser extent in the D.24-2 culture (only ∼50% reduction). Further, comparing this with the HSP expression data, an inverse correlation becomes apparent: culture D.HS-2 also shows the lowest activation of the cytosolic HSP70 isoforms, thus indicating that this culture was only undergoing a moderate heat-shock but shows the lowest reduction of SAg 51D transcripts.

As a consequence, SAg expression levels seem to be sensitive for heat-shocks which is supported by studies in the close relative *Tetrahymena thermophila,* where mRNA of variable surface antigens (*Ser* genes) was shown to be sensitive to heat-shocks; however, not necessarily because of physical instability but by active degradation by a *de novo* synthesized protein factor.^37,38^ This finding fits also very well to reports that a sudden change in temperature is much more efficient to induce serotype shifts compared with gradual changes.^10^

In conclusion, we found that many genes in the *Paramecium* genome show expression patterns that correlate with serotype expression. Their expression behaviour is characterized by high expression levels, certain stability to environmental changes and the sensitivity to heat-shocks (Fig. 6B).

As we know that heat-shocks induce serotype shifts more efficiently compared with more gradual changes and that the newly manifested serotype then becomes stabilized by epigenetic mechanisms, our finding that the entire spot of genes co-regulated with the SAg suggests that heat-shocks can induce widespread and long-term transcriptome alterations. In such case, the heat-shock could be interpreted as a kind of epigenetic reset on a large class of genes in the genome and would have an important influence on epigenetic memory.

### 3.9. Specific activation of SAgs in subtelomeric regions

**Figure 7.**
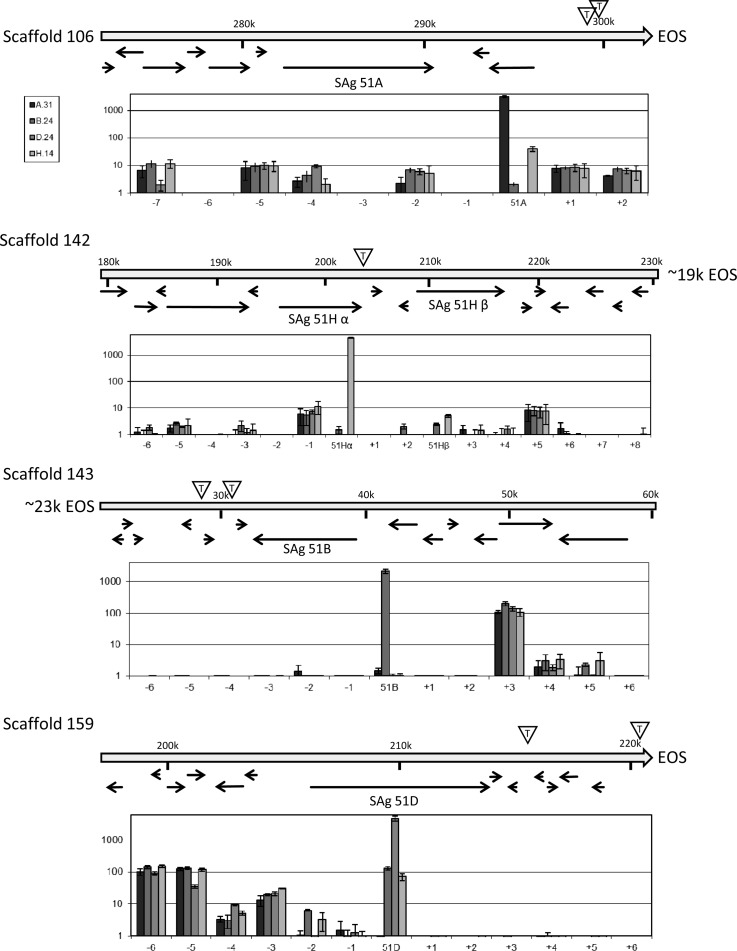
Genomic map and expression of SAg and neighbouring genes in subtelomeric regions. Genomic maps of subtelomeric regions of scaffold 106 (SAg 51A), scaffold 142 (SAg 51H), scaffold 143 (SAg 51B) and scaffold 159 (51D) indicate individual ORFs by arrows (data from *Paramecium*DB^22^ and Baranasic et al.^12^). The position in the scaffold is indicated above in kbp (k). Expression level of the SAg and nearby genes (numbered by –X upstream and +X downstream relative to the SAg) are shown by TPM normalized data (logarithmic scale) in the graph below the genomic maps, respectively. Internal telomeric sites representing chromosome heterogeneity are indicated by the triangle containing the letter ‘T’; EOS, end of scaffold.

A TPE was recently reported to control SAg expression and silencing by RNA interference in *P. tetraurelia*.^12^ The classical TPE is believed to silence genes by a spreading of the heterochromatic state from the telomere into the subtelomeric regions, and this is believed to be involved in the regulation of variant surface antigens in a series of pathogenic microbes.^39^ However, it remains unknown how individual genes avoid silencing and become thus expressed. To see whether the activation of an SAg goes along with a general activation of genes in this particular subtelomere, we investigated these loci more in detail. Figure 7 shows the subtelomeric surrounding of macronuclear chromosomes harbouring the here investigated SAgs 51A, 51B, 51D and 51H. The position of telomeres is indicated by EOS (end of scaffolds) as predicted by the genome project resulting in a majority of closed scaffolds with telomeric repeats on both ends^24^ or by the letter ‘T’ in a triangle indicating internal telomeric sites.^12^ These internal telomeric sites are due to the ∼800n polyploidy macronuclear chromosomes that exist in different length versions because of imprecise DNA elimination processes during maturation of small macronuclear chromosomes from larger micronuclear chromosomes.^40^ Figure 7 gives the combined information about gene position and expression levels of genes surrounding the respective SAg in all four serotype pure states 51A, 51B, 51D, 51H. The data do not indicate any events of spreading of heterochromatic silencing: on the one hand, activation of a subtelomeric SAg does not involve a loss of silencing of downstream genes (between SAg and telomere); on the other hand, the on/off state of the SAg does not apparently influence gene expression level of any neighbouring gene: their expression appears independent. As a conclusion, the TPE associated with SAg silencing and activation cannot be explained by a simple spreading of telomeric heterochromatin into subtelomeric regions, rather it seems more likely that the TPE influences SAg regulation in combination with a gene specific factor to specifically regulate one individual locus, thus avoiding global spreading events.

### 3.10. Conclusions

Cell differentiation is mainly discussed in context of the ability of stem cells to differentiate into different tissues, i.e. an epigenetic mechanism manifests an individual gene expression pattern of the genome. Are these mechanisms for regulating and manifesting gene expression the only difference to phenotypic variation of unicellular eukaryotes?

*Paramecium* was demonstrated in the past to differentiate its vegetative genome into different phenotypes with one prominent example in serotypes being regulated by epigenetically controlled heritable gene expression patterns whose regulation involves small RNA-induced chromatin regulation^12,13^ (Cheaib et al., in preparation). Interpretation of our transcriptome resource here indicates that not only the surface antigen multigene family but also many more genes follow the expression pattern of SAgs and show persistence while transcriptome adaptation to environmental changes. This suggests that gene expression patterns of gene groups can be manifested by epigenetic mechanisms and consequently that also unicellular species can differentiate into distinct epigenetically controlled phenotypes. However, such differentiation should be interpreted as a kind of transcriptomic robustness and needs to be distinguished from regular adaptation to environmental changes representing short-time flexibility for adaptation.

It seems clear that our study can only estimate the extent of epigenetic differentiation by the above-discussed mechanisms, and the high number of genes co-regulated with the SAgs justifies future approaches to clarify the epigenetic mechanisms controlling and maintaining gene expression. Of special interest here is the heat-shock, as the data indicate that this might represent a trigger for chromatin remodelling, not only to activate individual HSP genes but for remodelling many more chromosomal loci.

Our data suggest that serotypes cannot be restricted to the activation of a single gene as we identified a certain number of co-regulated genes, thus indicating that SAg expression is representative for individual gene expression profiles. *Paramecium* serotypes can be triggered by the environment or occur spontaneously (e.g. 51B cells in 51D cultures). It seems tempting to speculate that the co-regulated genes can be interpreted as a pre-installed genome program running to achieve a complex predetermined phenotype. We therefore need to clarify the epigenetic mechanisms allowing gene silencing, activation and maintenance of expression, to understand the epigenetic network that acts to coordinate the co-regulation of so many genes. Here, small RNA pathways, as indicated for the serotype system, would be an attractive hypothesis.

## Supplementary data

Supplementary data are available at www.dnaresearch.oxfordjournals.org.

## Funding

This study was supported by the starter grant by the Saarland University (61-cl/Anschub 2014/bew-Simon) and by grant SI 1397-2 by the Deutsche Forschungsgemeinschaft (DFG) to M.S. Funding to pay the Open Access publication charges for this article was provided by Saarland University.

## Supplementary Material

Supplementary Data
