# Supplementary material for: Epigenetic regulation of serotype expression antagonizes transcriptome dynamics in Paramecium tetraurelia
Source: DNA Res. 2015 Jul 31;22(4):293–305. doi: 10.1093/dnares/dsv014 (PMC4535620; doi:10.1093/dnares/dsv014)
Supplement: Supplementary Data [file supp_22_4_293__index.html]

Epigenetic regulation of serotype expression antagonizes transcriptome dynamics in Paramecium tetraurelia — Epigenetic regulation of serotype expression antagonizes transcriptome dynamics in Paramecium tetraurelia — Supplementary Data 

# Epigenetic regulation of serotype expression antagonizes transcriptome dynamics in *Paramecium tetraurelia*

## Supplementary Data

Supplementary Data

- Supplementary Data - Docx file
- Supplementary Data - txt file
- Supplementary Figure 1 - ppt file
- Supplementary Figure 2 - pdf file
- Supplementary Figure 3 - ppt file
- Supplementary Figure 4 - ppt file
- Supplementary Figure 5 - pdf file
