# Supplementary material for: Epigenetic regulation of serotype expression antagonizes transcriptome dynamics in Paramecium tetraurelia
Source: DNA Res. 2015 Jul 31;22(4):293–305. doi: 10.1093/dnares/dsv014 (PMC4535620; doi:10.1093/dnares/dsv014)
Supplement: Supplementary Data [file supp_dsv014_dsv014supp.docx]

# Supplementary files

### Suppl. Fig. 1 - Expression of developmentally regulated genes

The expression value of selected developmentally regulated genes based on TPM scaling is given to ensure that RNA was isolated from vegetative cultures. The time of expression of these genes during an autogamy time course (early-late) are indicated on the left according to reference^41^.

### Suppl. Fig. 2 - MA-plots of fold change expression level (y-Axis) against expression level (x-Axis).

Each point represents a transcript; those with significant differential expression (FDR below 0.01) are indicated in red.

**Suppl. Fig. 3 - Relationship between transcriptomes of cultures their replicates**

Clustering heat map showing expression levels (color key on the right: green high and blue low expression) obtained using Pearson correlation of log transformed normalized gene expression values.

### Suppl. Fig. 4 - Differentially expressed chromatin associated genes

(A) Lists of down- and upregulated chromatin associated genes between the B.24 vs. D.24 comparison. (B) Neighbor-joining tree of Histone H3 proteins of *Paramecium tetraurelia* (with 1000 bootstraps replicates) extracted from the *Paramecium*DB annotation^22^ except centromeric H3 isoforms. Expression data on autogamy specific activation were determined in a previous study by microarray analysis^41^. (C) The transcript quantification for the H3 isoforms is based on TMP normalization and shown in the table for all individual samples. Colour shading indicates relative expression level (dark blue-low; red-high).

### Suppl. File 5 - Lists with significantly differentially expressed GO terms

Lists of differentially expressed GO terms (FDR below 0.02).

**Suppl. File 6 - Sequence data of *Paramecium tetraurelia* HSP70 isoforms 11-16**

Multifasta file containing spliced cDNA sequences (cds) and amino acid sequences of the newly identified putative HSP70 isoforms.

**Reference**

41. Arnaiz, O., Gout, J.F., Betermier M., et al. 2010, Gene expression in a paleopolyploid: a transcriptome resource for the ciliate *Paramecium tetraurelia*, *BMC Genomics,* 11, 547.
