# Supplementary material for: Epigenetic regulation of serotype expression antagonizes transcriptome dynamics in Paramecium tetraurelia
Source: DNA Res. 2015 Jul 31;22(4):293–305. doi: 10.1093/dnares/dsv014 (PMC4535620; doi:10.1093/dnares/dsv014)
Supplement: Supplementary Data [file supp_dsv014_dsv014supp_fig1.ppt]

## Slide 1
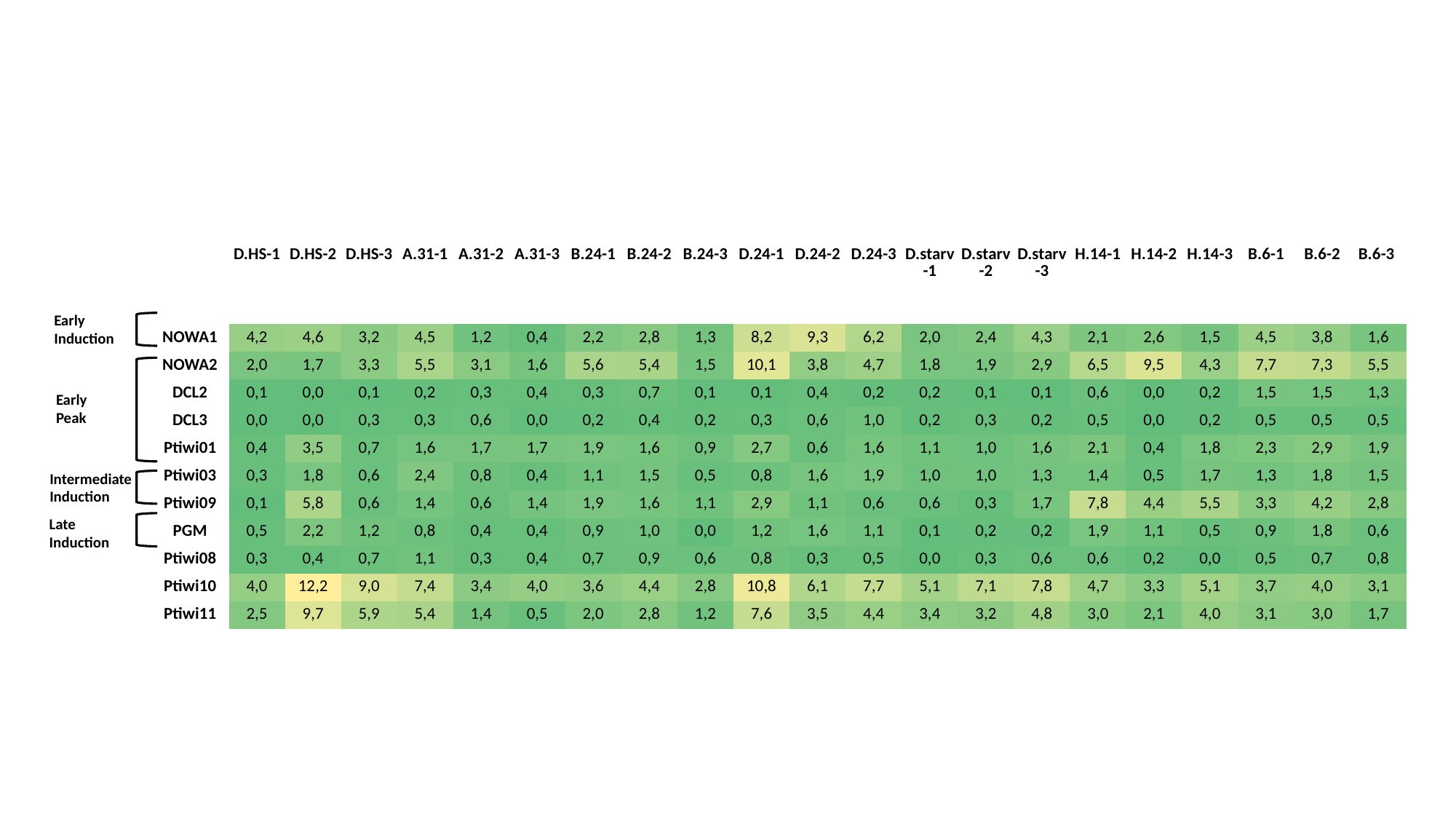

| | D.HS-1 | D.HS-2 | D.HS-3 | A.31-1 | A.31-2 | A.31-3 | B.24-1 | B.24-2 | B.24-3 | D.24-1 | D.24-2 | D.24-3 | D.starv-1 | D.starv-2 | D.starv-3 | H.14-1 | H.14-2 | H.14-3 | B.6-1 | B.6-2 | B.6-3 |
| --- | --- | --- | --- | --- | --- | --- | --- | --- | --- | --- | --- | --- | --- | --- | --- | --- | --- | --- | --- | --- | --- |
| | | | | | | | | | | | | | | | | | | | | | |
| NOWA1 | 4,2 | 4,6 | 3,2 | 4,5 | 1,2 | 0,4 | 2,2 | 2,8 | 1,3 | 8,2 | 9,3 | 6,2 | 2,0 | 2,4 | 4,3 | 2,1 | 2,6 | 1,5 | 4,5 | 3,8 | 1,6 |
| NOWA2 | 2,0 | 1,7 | 3,3 | 5,5 | 3,1 | 1,6 | 5,6 | 5,4 | 1,5 | 10,1 | 3,8 | 4,7 | 1,8 | 1,9 | 2,9 | 6,5 | 9,5 | 4,3 | 7,7 | 7,3 | 5,5 |
| DCL2 | 0,1 | 0,0 | 0,1 | 0,2 | 0,3 | 0,4 | 0,3 | 0,7 | 0,1 | 0,1 | 0,4 | 0,2 | 0,2 | 0,1 | 0,1 | 0,6 | 0,0 | 0,2 | 1,5 | 1,5 | 1,3 |
| DCL3 | 0,0 | 0,0 | 0,3 | 0,3 | 0,6 | 0,0 | 0,2 | 0,4 | 0,2 | 0,3 | 0,6 | 1,0 | 0,2 | 0,3 | 0,2 | 0,5 | 0,0 | 0,2 | 0,5 | 0,5 | 0,5 |
| Ptiwi01 | 0,4 | 3,5 | 0,7 | 1,6 | 1,7 | 1,7 | 1,9 | 1,6 | 0,9 | 2,7 | 0,6 | 1,6 | 1,1 | 1,0 | 1,6 | 2,1 | 0,4 | 1,8 | 2,3 | 2,9 | 1,9 |
| Ptiwi03 | 0,3 | 1,8 | 0,6 | 2,4 | 0,8 | 0,4 | 1,1 | 1,5 | 0,5 | 0,8 | 1,6 | 1,9 | 1,0 | 1,0 | 1,3 | 1,4 | 0,5 | 1,7 | 1,3 | 1,8 | 1,5 |
| Ptiwi09 | 0,1 | 5,8 | 0,6 | 1,4 | 0,6 | 1,4 | 1,9 | 1,6 | 1,1 | 2,9 | 1,1 | 0,6 | 0,6 | 0,3 | 1,7 | 7,8 | 4,4 | 5,5 | 3,3 | 4,2 | 2,8 |
| PGM | 0,5 | 2,2 | 1,2 | 0,8 | 0,4 | 0,4 | 0,9 | 1,0 | 0,0 | 1,2 | 1,6 | 1,1 | 0,1 | 0,2 | 0,2 | 1,9 | 1,1 | 0,5 | 0,9 | 1,8 | 0,6 |
| Ptiwi08 | 0,3 | 0,4 | 0,7 | 1,1 | 0,3 | 0,4 | 0,7 | 0,9 | 0,6 | 0,8 | 0,3 | 0,5 | 0,0 | 0,3 | 0,6 | 0,6 | 0,2 | 0,0 | 0,5 | 0,7 | 0,8 |
| Ptiwi10 | 4,0 | 12,2 | 9,0 | 7,4 | 3,4 | 4,0 | 3,6 | 4,4 | 2,8 | 10,8 | 6,1 | 7,7 | 5,1 | 7,1 | 7,8 | 4,7 | 3,3 | 5,1 | 3,7 | 4,0 | 3,1 |
| Ptiwi11 | 2,5 | 9,7 | 5,9 | 5,4 | 1,4 | 0,5 | 2,0 | 2,8 | 1,2 | 7,6 | 3,5 | 4,4 | 3,4 | 3,2 | 4,8 | 3,0 | 2,1 | 4,0 | 3,1 | 3,0 | 1,7 |
Early
Induction
Early
Peak
Intermediate
Induction
Late
Induction
