# Supplementary material for: Epigenetic regulation of serotype expression antagonizes transcriptome dynamics in Paramecium tetraurelia
Source: DNA Res. 2015 Jul 31;22(4):293–305. doi: 10.1093/dnares/dsv014 (PMC4535620; doi:10.1093/dnares/dsv014)
Supplement: Supplementary Data [file supp_dsv014_dsv014supp_fig2.pdf]

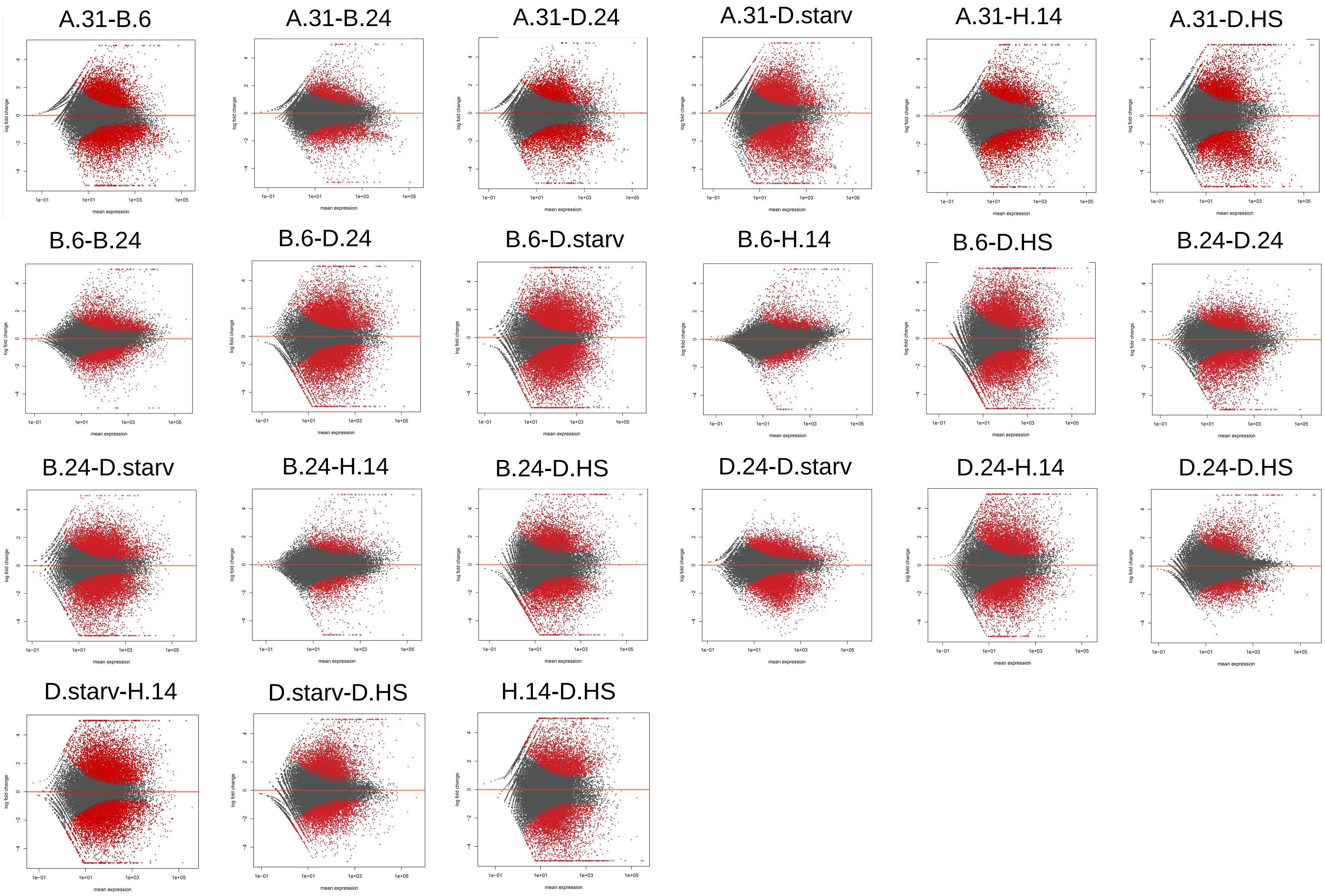
A.31-B.6
A.31-B.24
A.31-D.24
A.31-D.starv
A.31-H.14
A.31-D.HS
B.6-B.24
B.6-D.24
B.6-D.starv
B.6-H.14
B.6-D.HS
B.24-D.24
B.24-D.starv
B.24-H.14
B.24-D.HS
D.24-D.starv
D.24-H.14
D.24-D.HS
D.starv-H.14
D.starv-D.HS
H.14-D.HS
log fold change
mean expression
1e-01
1e+01
1e+03
1e+05
