# Supplementary figures and images for: Epigenetic regulation of serotype expression antagonizes transcriptome dynamics in Paramecium tetraurelia
Source: DNA Res. 2015 Jul 31;22(4):293–305. doi: 10.1093/dnares/dsv014 (PMC4535620; doi:10.1093/dnares/dsv014)

## Slide 1
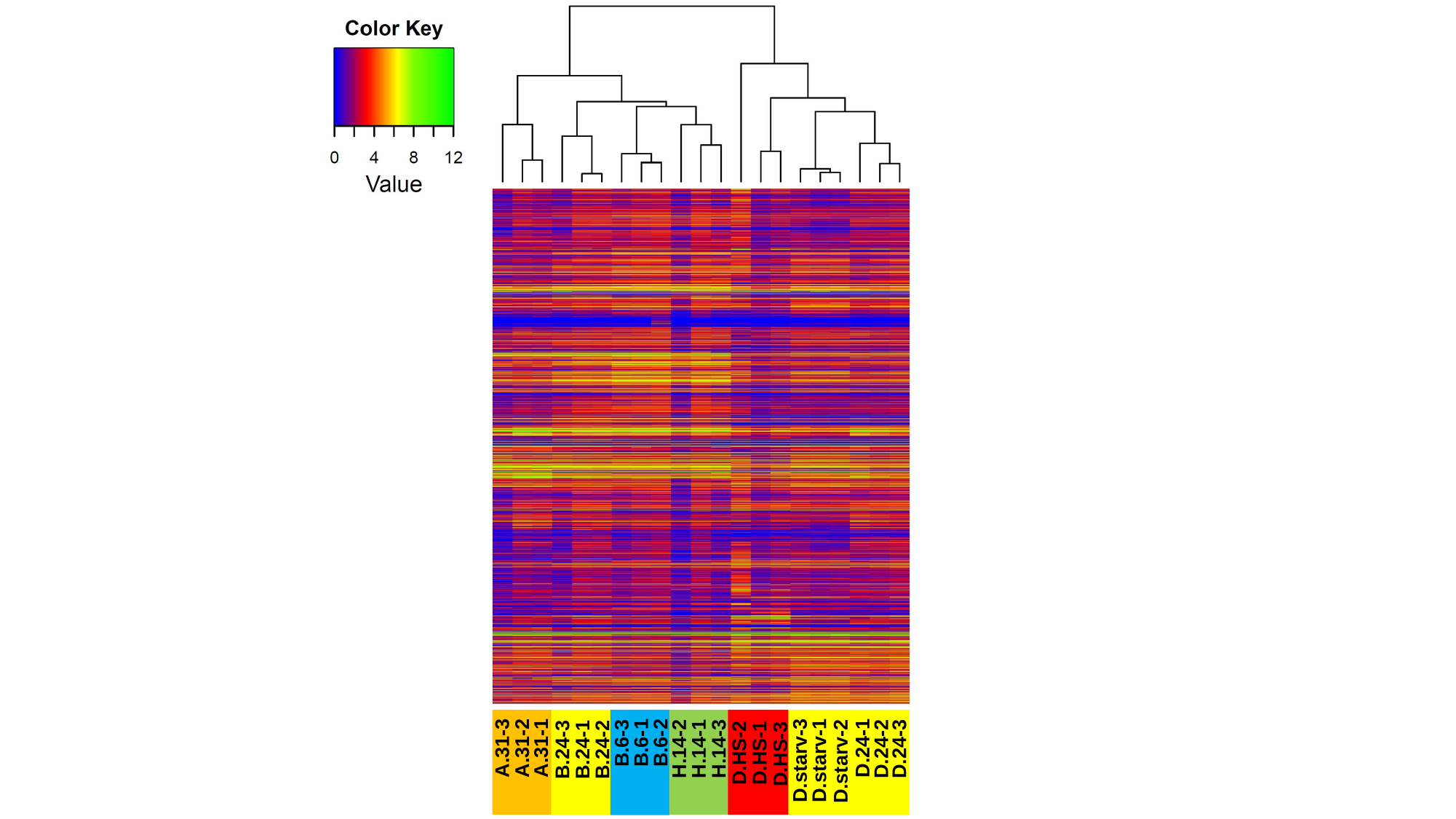

B.6-3
B.6-1
B.6-2
D.24-1
A.31-3
A.31-2
A.31-1
H.14-2
H.14-1
H.14-3
D.24-2
D.24-3
B.24-3
B.24-1
B.24-2
D.HS-2
D.HS-1
D.HS-3
D.starv-3
D.starv-1
D.starv-2

Supplement: Supplementary Data [file supp_dsv014_dsv014supp_fig3.ppt]
