# Supplementary material for: Epigenetic regulation of serotype expression antagonizes transcriptome dynamics in Paramecium tetraurelia
Source: DNA Res. 2015 Jul 31;22(4):293–305. doi: 10.1093/dnares/dsv014 (PMC4535620; doi:10.1093/dnares/dsv014)
Supplement: Supplementary Data [file supp_dsv014_dsv014supp_fig4.ppt]

## Slide 1
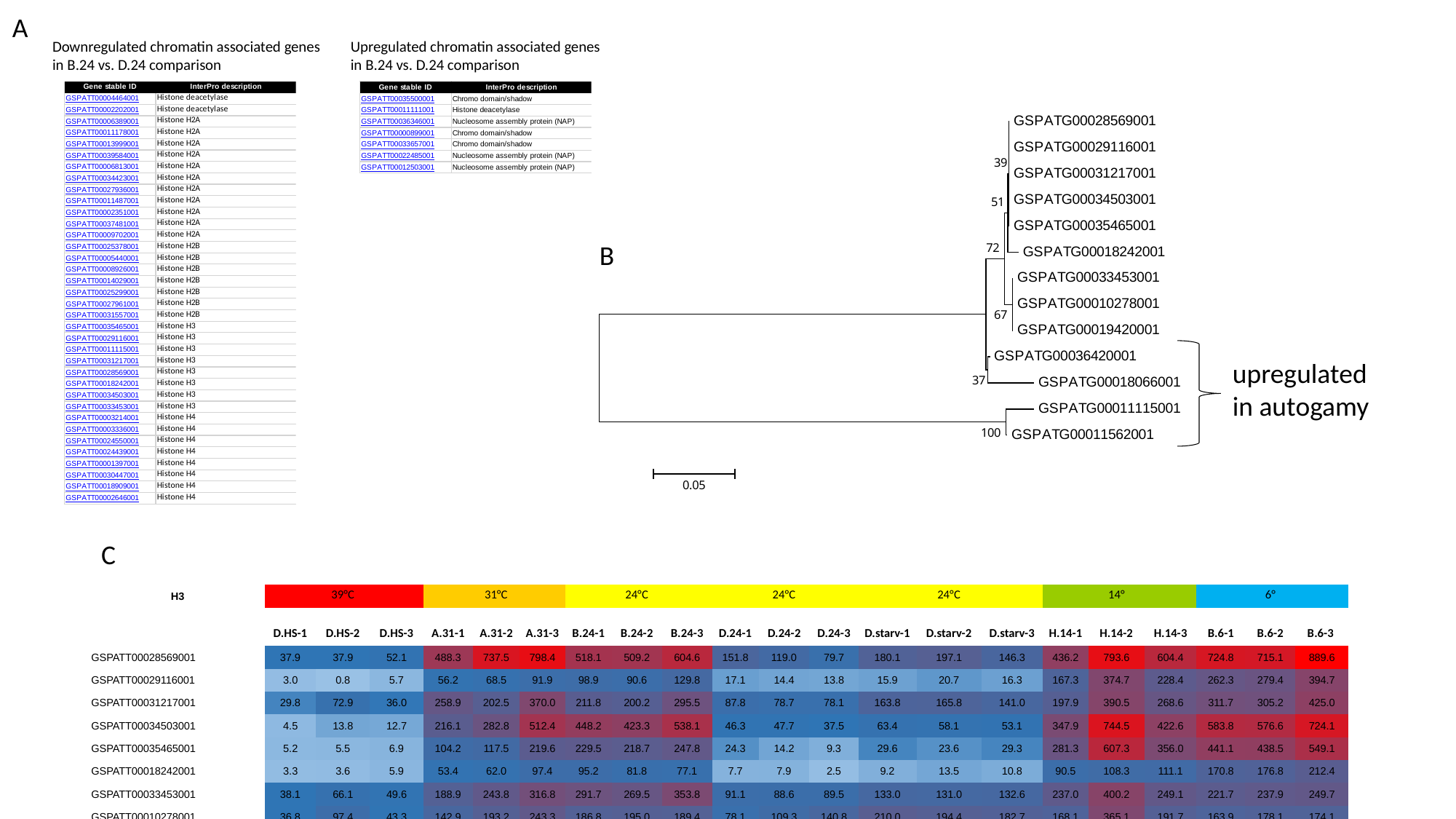

A
Downregulated chromatin associated genes
in B.24 vs. D.24 comparison
Upregulated chromatin associated genes
in B.24 vs. D.24 comparison
B
upregulated
in autogamy
C
| H3 | | 39°C | | | 31°C | | | 24°C | | | 24°C | | | 24°C | | | 14° | | | 6° | |
| --- | --- | --- | --- | --- | --- | --- | --- | --- | --- | --- | --- | --- | --- | --- | --- | --- | --- | --- | --- | --- | --- |
| | D.HS-1 | D.HS-2 | D.HS-3 | A.31-1 | A.31-2 | A.31-3 | B.24-1 | B.24-2 | B.24-3 | D.24-1 | D.24-2 | D.24-3 | D.starv-1 | D.starv-2 | D.starv-3 | H.14-1 | H.14-2 | H.14-3 | B.6-1 | B.6-2 | B.6-3 |
| GSPATT00028569001 | 37.9 | 37.9 | 52.1 | 488.3 | 737.5 | 798.4 | 518.1 | 509.2 | 604.6 | 151.8 | 119.0 | 79.7 | 180.1 | 197.1 | 146.3 | 436.2 | 793.6 | 604.4 | 724.8 | 715.1 | 889.6 |
| GSPATT00029116001 | 3.0 | 0.8 | 5.7 | 56.2 | 68.5 | 91.9 | 98.9 | 90.6 | 129.8 | 17.1 | 14.4 | 13.8 | 15.9 | 20.7 | 16.3 | 167.3 | 374.7 | 228.4 | 262.3 | 279.4 | 394.7 |
| GSPATT00031217001 | 29.8 | 72.9 | 36.0 | 258.9 | 202.5 | 370.0 | 211.8 | 200.2 | 295.5 | 87.8 | 78.7 | 78.1 | 163.8 | 165.8 | 141.0 | 197.9 | 390.5 | 268.6 | 311.7 | 305.2 | 425.0 |
| GSPATT00034503001 | 4.5 | 13.8 | 12.7 | 216.1 | 282.8 | 512.4 | 448.2 | 423.3 | 538.1 | 46.3 | 47.7 | 37.5 | 63.4 | 58.1 | 53.1 | 347.9 | 744.5 | 422.6 | 583.8 | 576.6 | 724.1 |
| GSPATT00035465001 | 5.2 | 5.5 | 6.9 | 104.2 | 117.5 | 219.6 | 229.5 | 218.7 | 247.8 | 24.3 | 14.2 | 9.3 | 29.6 | 23.6 | 29.3 | 281.3 | 607.3 | 356.0 | 441.1 | 438.5 | 549.1 |
| GSPATT00018242001 | 3.3 | 3.6 | 5.9 | 53.4 | 62.0 | 97.4 | 95.2 | 81.8 | 77.1 | 7.7 | 7.9 | 2.5 | 9.2 | 13.5 | 10.8 | 90.5 | 108.3 | 111.1 | 170.8 | 176.8 | 212.4 |
| GSPATT00033453001 | 38.1 | 66.1 | 49.6 | 188.9 | 243.8 | 316.8 | 291.7 | 269.5 | 353.8 | 91.1 | 88.6 | 89.5 | 133.0 | 131.0 | 132.6 | 237.0 | 400.2 | 249.1 | 221.7 | 237.9 | 249.7 |
| GSPATT00010278001 | 36.8 | 97.4 | 43.3 | 142.9 | 193.2 | 243.3 | 186.8 | 195.0 | 189.4 | 78.1 | 109.3 | 140.8 | 210.0 | 194.4 | 182.7 | 168.1 | 365.1 | 191.7 | 163.9 | 178.1 | 174.1 |
| GSPATT00019420001 | 13.9 | 61.2 | 18.5 | 76.6 | 93.4 | 157.6 | 95.9 | 100.0 | 101.4 | 72.1 | 91.7 | 120.6 | 146.9 | 172.6 | 145.3 | 96.7 | 125.3 | 113.9 | 141.3 | 126.8 | 155.7 |
| GSPATT00036420001 | 0.2 | 0.0 | 0.8 | 10.6 | 9.6 | 15.3 | 4.2 | 4.6 | 3.4 | 7.6 | 0.6 | 0.4 | 1.5 | 2.3 | 2.5 | 11.5 | 12.4 | 13.4 | 28.6 | 33.2 | 37.3 |
| GSPATT00018066001 | 0.0 | 0.5 | 1.3 | 2.9 | 1.2 | 2.8 | 0.0 | 0.3 | 0.1 | 0.7 | 0.2 | 0.0 | 0.3 | 0.8 | 0.1 | 0.7 | 0.9 | 2.5 | 0.7 | 1.1 | 1.0 |
| GSPATT00011115001 | 1.7 | 2.4 | 1.2 | 3.4 | 3.5 | 7.0 | 14.0 | 11.4 | 6.7 | 0.9 | 1.3 | 0.4 | 3.6 | 2.4 | 2.3 | 30.5 | 32.5 | 40.3 | 30.4 | 34.8 | 38.3 |
| GSPATT00011562001 | 0.3 | 0.0 | 0.4 | 6.8 | 2.4 | 2.6 | 6.8 | 6.2 | 4.6 | 4.1 | 0.6 | 2.5 | 0.8 | 0.7 | 1.2 | 13.4 | 7.7 | 11.0 | 12.6 | 20.4 | 22.0 |
