# Supplementary material for: Epigenetic regulation of serotype expression antagonizes transcriptome dynamics in Paramecium tetraurelia
Source: DNA Res. 2015 Jul 31;22(4):293–305. doi: 10.1093/dnares/dsv014 (PMC4535620; doi:10.1093/dnares/dsv014)
Supplement: Supplementary Data [file supp_dsv014_dsv014supp_fig5.pdf]

D.24-D.HS

Downregulated

ID	p.adjusted	Biological function
GO:0032502	3.19E-19	developmental process
GO:0032501	9.91E-19	multicellular organismal process
GO:0006508	4.08E-16	proteolysis
GO:0044767	6.02E-16	single-organism developmental process
GO:0044707	2.23E-15	single-multicellular organism process
GO:0016020	5.14E-15	membrane
GO:0016787	6.01E-11	hydrolase activity
GO:0071806	5.25E-10	protein transmembrane transport
GO:0008233	5.79E-10	peptidase activity
GO:0022884	8.40E-09	macromolecule transmembrane transporter activity
GO:0009306	3.58E-08	protein secretion
GO:0008061	4.22E-08	chitin binding
GO:0015450	4.72E-08	PP-bond-hydrolysis-driven protein transmembrane transporter activity
GO:0008320	9.99E-08	protein transmembrane transporter activity
GO:0055085	1.74E-07	transmembrane transport
GO:0005215	2.18E-07	transporter activity
GO:0060089	1.81E-06	molecular transducer activity
GO:0006022	1.02E-05	aminoglycan metabolic process
GO:0044699	2.40E-05	single-organism process
GO:0032940	3.01E-05	secretion by cell
GO:0006040	8.76E-05	amino sugar metabolic process
GO:0008565	6.17E-04	protein transporter activity
GO:0005576	8.68E-04	extracellular region
GO:0005575	1.83E-03	cellular_component
GO:0046903	3.06E-03	secretion
GO:0006605	5.04E-03	protein targeting
GO:0044765	7.35E-03	single-organism transport
GO:0008150	7.35E-03	biological_process
GO:0030246	7.35E-03	carbohydrate binding
GO:1902578	8.10E-03	single-organism localization
GO:0048869	8.99E-03	cellular developmental process
GO:0051179	9.41E-03	localization

Upregulated

ID	p.adjusted	Biological function
GO:0006457	7.3E-04	protein folding
GO:2000112	7.3E-04	regulation of cellular macromolecule biosynthetic process
GO:0010556	7.3E-04	regulation of macromolecule biosynthetic process
GO:0005488	7.3E-04	binding
GO:0032774	8.4E-04	RNA biosynthetic process
GO:0006351	1.1E-03	transcription, DNA-templated
GO:0031326	1.4E-03	regulation of cellular biosynthetic process
GO:0043227	2.5E-03	membrane-bounded organelle
GO:0043231	2.5E-03	intracellular membrane-bounded organelle
GO:0031072	2.7E-03	heat shock protein binding
GO:0046914	3.3E-03	transition metal ion binding
GO:0010468	3.3E-03	regulation of gene expression
GO:0044271	3.6E-03	cellular nitrogen compound biosynthetic process
GO:1901362	9.9E-03	organic cyclic compound biosynthetic process
GO:0005515	9.9E-03	protein binding

GO:0018130	1.1E-02	heterocycle biosynthetic process
GO:0009889	1.5E-02	regulation of biosynthetic process
GO:0051082	1.6E-02	unfolded protein binding

D.24-D.starv

Downregulated

**ID**	**p.adjusted**	**Biological function**
GO:0005856	1.89E-24	cytoskeleton
GO:0006928	1.14E-22	movement of cell or subcellular component
GO:0005875	6.93E-22	microtubule associated complex
GO:0003774	4.90E-19	motor activity
GO:0007017	1.67E-17	microtubule-based process
GO:0044430	9.48E-14	cytoskeletal part
GO:0005509	1.40E-12	calcium ion binding
GO:0043234	3.51E-11	protein complex
GO:0030286	5.94E-09	dynein complex
GO:0044446	5.33E-08	intracellular organelle part
GO:0044422	1.20E-07	organelle part
GO:0072511	2.29E-06	divalent inorganic cation transport
GO:0016817	4.29E-06	hydrolase activity, acting on acid anhydrides
GO:0048500	5.55E-06	signal recognition particle
GO:0072509	6.88E-06	divalent inorganic cation transmembrane transporter activity
GO:0006614	2.72E-05	SRP-dependent cotranslational protein targeting to membrane
GO:0030001	4.06E-05	metal ion transport
GO:0070588	4.21E-05	calcium ion transmembrane transport
GO:1901265	7.36E-05	nucleoside phosphate binding
GO:0045047	7.36E-05	protein targeting to ER
GO:0006418	7.36E-05	tRNA aminoacylation for protein translation
GO:0012506	1.25E-04	vesicle membrane
GO:0016675	1.37E-04	oxidoreductase activity, acting on a heme group of donors
GO:0015002	1.37E-04	heme-copper terminal oxidase activity
GO:0004129	1.37E-04	cytochrome-c oxidase activity
GO:0004175	1.78E-04	endopeptidase activity
GO:0032502	1.99E-04	developmental process
GO:0009306	2.23E-04	protein secretion
GO:0070647	3.36E-04	protein modification by small protein conjugation or removal
GO:0007018	5.24E-04	microtubule-based movement
GO:0008312	5.30E-04	7S RNA binding
GO:0032501	5.67E-04	multicellular organismal process
GO:0019205	6.12E-04	nucleobase-containing compound kinase activity
GO:0009073	7.33E-04	aromatic amino acid family biosynthetic process
GO:0046873	7.77E-04	metal ion transmembrane transporter activity
GO:0031424	8.84E-04	keratinization
GO:0044767	9.42E-04	single-organism developmental process
GO:0044763	1.03E-03	single-organism cellular process
GO:0016192	1.15E-03	vesicle-mediated transport
GO:0005216	1.22E-03	ion channel activity
GO:0031988	1.22E-03	membrane-bounded vesicle
GO:0016023	1.22E-03	cytoplasmic membrane-bounded vesicle
GO:0030855	1.33E-03	epithelial cell differentiation
GO:0005488	1.43E-03	binding
GO:0044707	2.27E-03	single-multicellular organism process
GO:0006605	2.43E-03	protein targeting
GO:0019787	2.47E-03	ubiquitin-like protein transferase activity
GO:0016874	2.47E-03	ligase activity

GO:0005524	3.52E-03	ATP binding
GO:0032559	3.52E-03	adenyl ribonucleotide binding
GO:0030554	3.52E-03	adenyl nucleotide binding
GO:0015085	3.73E-03	calcium ion transmembrane transporter activity
GO:0090150	3.74E-03	establishment of protein localization to membrane
GO:0070838	3.98E-03	divalent metal ion transport
GO:0072657	4.40E-03	protein localization to membrane
GO:0015450	5.48E-03	PP-bond-hydrolysis-driven protein transmembrane transporter activity
GO:0006508	6.81E-03	proteolysis
GO:0009190	7.05E-03	cyclic nucleotide biosynthetic process
GO:0022857	7.51E-03	transmembrane transporter activity
GO:0016787	1.07E-02	hydrolase activity
GO:0022884	1.09E-02	macromolecule transmembrane transporter activity
GO:0031982	1.11E-02	vesicle
GO:0071806	1.24E-02	protein transmembrane transport
GO:0072599	1.24E-02	establishment of protein localization to endoplasmic reticulum
GO:0019932	1.25E-02	second-messenger-mediated signaling
GO:0031410	1.25E-02	cytoplasmic vesicle
GO:0004674	1.26E-02	protein serine/threonine kinase activity
GO:0043228	1.44E-02	non-membrane-bounded organelle
GO:0044765	1.46E-02	single-organism transport
GO:0001882	1.53E-02	nucleoside binding
GO:0030662	1.56E-02	coated vesicle membrane
GO:0044433	1.61E-02	cytoplasmic vesicle part
GO:0022803	1.61E-02	passive transmembrane transporter activity
GO:0022838	1.61E-02	substrate-specific channel activity
GO:0008320	1.83E-02	protein transmembrane transporter activity
GO:1902578	1.84E-02	single-organism localization

Upregulated

**ID**	**p.adjusted**	**Biological function**
GO:0006629	9.00E-06	lipid metabolic process
GO:0052689	9.00E-06	carboxylic ester hydrolase activity
GO:0019866	1.07E-05	organelle inner membrane
GO:0016298	1.16E-05	lipase activity
GO:0044429	1.00E-04	mitochondrial part
GO:0032993	1.00E-04	protein-DNA complex
GO:0044815	1.41E-04	DNA packaging complex
GO:1990104	1.41E-04	DNA bending complex
GO:0005739	5.09E-04	mitochondrion
GO:0000785	1.02E-03	chromatin
GO:0016020	1.02E-03	membrane
GO:0031967	1.02E-03	organelle envelope
GO:0030029	1.02E-03	actin filament-based process
GO:0044710	1.09E-03	single-organism metabolic process
GO:0031975	1.19E-03	envelope
GO:0006508	2.18E-03	proteolysis
GO:0008654	2.44E-03	phospholipid biosynthetic process
GO:0006308	2.44E-03	DNA catabolic process
GO:0008301	2.64E-03	DNA binding, bending
GO:1901617	3.77E-03	organic hydroxy compound biosynthetic process
GO:0008610	4.82E-03	lipid biosynthetic process
GO:0006996	4.86E-03	organelle organization
GO:0006644	5.71E-03	phospholipid metabolic process
GO:0006020	6.04E-03	inositol metabolic process

GO:0045454	6.32E-03	cell redox homeostasis
GO:0034655	7.53E-03	nucleobase-containing compound catabolic process
GO:0043687	7.53E-03	post-translational protein modification
GO:0031966	9.04E-03	mitochondrial membrane
GO:0019439	9.51E-03	aromatic compound catabolic process
GO:0005694	9.57E-03	chromosome
GO:0008233	9.60E-03	peptidase activity
GO:0044270	1.03E-02	cellular nitrogen compound catabolic process
GO:0018208	1.10E-02	peptidyl-proline modification
GO:0046914	1.16E-02	transition metal ion binding
GO:0030041	1.16E-02	actin filament polymerization
GO:0006323	1.18E-02	DNA packaging
GO:0046700	1.20E-02	heterocycle catabolic process
GO:0043227	1.26E-02	membrane-bounded organelle
GO:0043231	1.33E-02	intracellular membrane-bounded organelle
GO:0044427	1.38E-02	chromosomal part
GO:0016491	1.57E-02	oxidoreductase activity
GO:0019725	1.62E-02	cellular homeostasis
GO:0090407	1.84E-02	organophosphate biosynthetic process
GO:1901361	1.84E-02	organic cyclic compound catabolic process

B.24-D.24

Downregulated

**ID**	**p.adjusted**	**Biological function**
GO:0016491	9.16E-34	oxidoreductase activity
GO:0008301	6.37E-23	DNA binding, bending
GO:0032993	2.97E-22	protein-DNA complex
GO:0044710	2.97E-22	single-organism metabolic process
GO:0044815	7.07E-22	DNA packaging complex
GO:1990104	7.07E-22	DNA bending complex
GO:0048037	4.79E-21	cofactor binding
GO:0006082	7.22E-21	organic acid metabolic process
GO:0006323	1.32E-18	DNA packaging
GO:0031497	5.15E-17	chromatin assembly
GO:0071824	6.80E-17	protein-DNA complex subunit organization
GO:0055114	8.97E-17	oxidation-reduction process
GO:0034728	1.00E-16	nucleosome organization
GO:0050660	6.91E-15	flavin adenine dinucleotide binding
GO:0006334	2.91E-13	nucleosome assembly
GO:0065004	2.17E-12	protein-DNA complex assembly
GO:0000786	2.49E-11	nucleosome
GO:0000785	8.27E-11	chromatin
GO:0006091	1.24E-10	generation of precursor metabolites and energy
GO:0044424	1.62E-10	intracellular part
GO:0044427	4.14E-10	chromosomal part
GO:0008150	1.19E-08	biological_process
GO:0005975	1.24E-08	carbohydrate metabolic process
GO:0005694	4.89E-08	chromosome
GO:0003824	8.85E-08	catalytic activity
GO:0006325	8.85E-08	chromatin organization
GO:1901575	2.60E-07	organic substance catabolic process
GO:0008233	1.53E-06	peptidase activity
GO:0006096	1.57E-06	glycolytic process
GO:0042579	2.13E-06	microbody
GO:0009056	2.53E-06	catabolic process

GO:0043226	2.57E-06	organelle
GO:1901564	3.86E-06	organonitrogen compound metabolic process
GO:0071103	4.25E-06	DNA conformation change
GO:0032991	6.24E-06	macromolecular complex
GO:0006996	6.24E-06	organelle organization
GO:0044242	8.21E-06	cellular lipid catabolic process
GO:0006520	2.06E-05	cellular amino acid metabolic process
GO:0044262	2.52E-05	cellular carbohydrate metabolic process
GO:0019842	4.70E-05	vitamin binding
GO:0022607	6.40E-05	cellular component assembly
GO:0016769	7.18E-05	transferase activity, transferring nitrogenous groups
GO:0045454	8.29E-05	cell redox homeostasis
GO:0006412	1.02E-04	translation
GO:0009124	1.05E-04	nucleoside monophosphate biosynthetic process
GO:0006470	1.09E-04	protein dephosphorylation
GO:0044422	1.09E-04	organelle part
GO:0008519	1.61E-04	ammonium transmembrane transporter activity
GO:0098661	1.96E-04	inorganic anion transmembrane transport
GO:0030976	2.14E-04	thiamine pyrophosphate binding
GO:0098660	2.14E-04	inorganic ion transmembrane transport
GO:0098662	2.14E-04	inorganic cation transmembrane transport
GO:0016757	2.54E-04	transferase activity, transferring glycosyl groups
GO:0000502	2.90E-04	proteasome complex
GO:0009123	3.10E-04	nucleoside monophosphate metabolic process
GO:0034440	3.10E-04	lipid oxidation
GO:0015980	3.24E-04	energy derivation by oxidation of organic compounds
GO:1901135	3.44E-04	carbohydrate derivative metabolic process
GO:0015695	3.53E-04	organic cation transport
GO:0072488	3.80E-04	ammonium transmembrane transport
GO:0006818	4.28E-04	hydrogen transport
GO:0019693	4.37E-04	ribose phosphate metabolic process
GO:0005839	4.37E-04	proteasome core complex
GO:0065003	4.37E-04	macromolecular complex assembly
GO:0071705	4.37E-04	nitrogen compound transport
GO:1901681	4.37E-04	sulfur compound binding
GO:0030332	4.41E-04	cyclin binding
GO:0044085	4.75E-04	cellular component biogenesis
GO:0006508	5.05E-04	proteolysis
GO:0005575	6.75E-04	cellular_component
GO:0009055	7.38E-04	electron carrier activity
GO:0072522	7.83E-04	purine-containing compound biosynthetic process
GO:0051726	8.48E-04	regulation of cell cycle
GO:0097472	1.05E-03	cyclin-dependent protein kinase activity
GO:0071822	1.29E-03	protein complex subunit organization
GO:0004693	1.55E-03	cyclin-dependent protein serine/threonine kinase activity
GO:0044712	1.55E-03	single-organism catabolic process
GO:0006812	1.65E-03	cation transport
GO:0004298	1.66E-03	threonine-type endopeptidase activity
GO:0015101	1.66E-03	organic cation transmembrane transporter activity
GO:0065008	1.75E-03	regulation of biological quality
GO:0070003	1.75E-03	threonine-type peptidase activity
GO:0005506	1.93E-03	iron ion binding
GO:1901137	1.93E-03	carbohydrate derivative biosynthetic process
GO:0008509	2.05E-03	anion transmembrane transporter activity
GO:0044255	2.35E-03	cellular lipid metabolic process
GO:0016538	2.37E-03	cyclin-dependent protein serine/threonine kinase regulator activity

GO:1902600	2.37E-03	hydrogen ion transmembrane transport
GO:0043227	3.07E-03	membrane-bounded organelle
GO:0016311	3.09E-03	dephosphorylation
GO:0004674	3.35E-03	protein serine/threonine kinase activity
GO:0043231	3.45E-03	intracellular membrane-bounded organelle
GO:0046912	3.76E-03	transferase activity, transferring acyl groups
GO:0098656	3.80E-03	anion transmembrane transport
GO:0006631	4.09E-03	fatty acid metabolic process
GO:0009163	4.83E-03	nucleoside biosynthetic process
GO:0006414	5.36E-03	translational elongation
GO:0015696	5.48E-03	ammonium transport
GO:0030163	6.05E-03	protein catabolic process
GO:0043413	6.69E-03	macromolecule glycosylation
GO:0019395	6.77E-03	fatty acid oxidation
GO:0051287	7.53E-03	NAD binding
GO:0006730	8.63E-03	one-carbon metabolic process
GO:0030337	9.30E-03	DNA polymerase processivity factor activity
GO:0003677	1.06E-02	DNA binding
GO:0051540	1.12E-02	metal cluster binding
GO:0046034	1.12E-02	ATP metabolic process
GO:0072329	1.15E-02	monocarboxylic acid catabolic process
GO:0006629	1.16E-02	lipid metabolic process
GO:0098589	1.17E-02	membrane region
GO:0016042	1.22E-02	lipid catabolic process
GO:0009142	1.36E-02	nucleoside triphosphate biosynthetic process
GO:0016884	1.44E-02	carbon-nitrogen ligase activity, with glutamine as amido-N-donor
GO:0006486	1.44E-02	protein glycosylation
GO:0003735	1.44E-02	structural constituent of ribosome
GO:0006164	1.46E-02	purine nucleotide biosynthetic process
GO:0006119	1.46E-02	oxidative phosphorylation
GO:0098655	1.47E-02	cation transmembrane transport
GO:0008237	1.61E-02	metallopeptidase activity
GO:0005789	1.63E-02	endoplasmic reticulum membrane
GO:0006754	1.67E-02	ATP biosynthetic process
GO:0030170	1.67E-02	pyridoxal phosphate binding
GO:0005623	1.67E-02	cell
GO:0044464	1.67E-02	cell part
GO:0032787	1.69E-02	monocarboxylic acid metabolic process
GO:0042175	1.70E-02	nuclear outer membrane-endoplasmic reticulum membrane network
GO:0009141	1.73E-02	nucleoside triphosphate metabolic process
GO:0042625	1.77E-02	ATPase activity, coupled to transmembrane movement of ions
GO:0072521	1.83E-02	purine-containing compound metabolic process
GO:0009062	1.91E-02	fatty acid catabolic process
GO:0016758	1.91E-02	transferase activity, transferring hexosyl groups

Upregulated

**ID**	**p.adjusted**	**Biological function**
GO:0051082	1.5E-04	unfolded protein binding
GO:0006457	1.2E-02	protein folding
GO:0043168	2.6E-02	anion binding
GO:0003674	2.6E-02	molecular_function
GO:0005853	2.6E-02	eukaryotic translation elongation factor 1 complex
GO:0005488	3.9E-02	binding
GO:0048870	5.8E-02	cell motility
GO:0006468	5.8E-02	protein phosphorylation

GO:0051674	7.2E-02	localization of cell
GO:0005952	7.2E-02	cAMP-dependent protein kinase complex
GO:0016772	7.2E-02	transferase activity, transferring phosphorus-containing groups
GO:0006793	7.2E-02	phosphorus metabolic process
GO:0006418	7.2E-02	tRNA aminoacylation for protein translation
GO:0005984	7.2E-02	disaccharide metabolic process
GO:0072509	7.2E-02	divalent inorganic cation transmembrane transporter activity
GO:0018193	7.3E-02	peptidyl-amino acid modification
GO:0008135	7.9E-02	translation factor activity, nucleic acid binding
GO:0016272	7.9E-02	prefoldin complex
GO:0097367	9.4E-02	carbohydrate derivative binding
GO:0016051	1.2E-01	carbohydrate biosynthetic process
GO:0044267	1.5E-01	cellular protein metabolic process
GO:0005388	1.6E-01	calcium-transporting ATPase activity
GO:0009288	1.6E-01	bacterial-type flagellum
GO:1901363	1.6E-01	heterocyclic compound binding
GO:0097159	1.6E-01	organic cyclic compound binding
GO:0036094	1.6E-01	small molecule binding
GO:0003746	1.6E-01	translation elongation factor activity
GO:0046434	1.6E-01	organophosphate catabolic process
GO:0040011	1.7E-01	locomotion
GO:0009311	1.7E-01	oligosaccharide metabolic process
GO:0032312	1.9E-01	regulation of ARF GTPase activity
GO:0072511	1.9E-01	divalent inorganic cation transport

B.6-B.24

Downregulated

**ID**	**p.adjusted**	**Biological function**
GO:0006259	5.9E-16	DNA metabolic process
GO:0006260	1.9E-14	DNA replication
GO:0071897	2.0E-14	DNA biosynthetic process
GO:0006725	6.4E-14	cellular aromatic compound metabolic process
GO:0034641	4.3E-13	cellular nitrogen compound metabolic process
GO:0046483	8.0E-13	heterocycle metabolic process
GO:0090304	2.3E-12	nucleic acid metabolic process
GO:1901360	8.4E-12	organic cyclic compound metabolic process
GO:0006139	1.4E-10	nucleobase-containing compound metabolic process
GO:0043231	1.1E-07	intracellular membrane-bounded organelle
GO:0016887	1.8E-07	ATPase activity
GO:0043227	1.9E-07	membrane-bounded organelle
GO:0006807	3.5E-07	nitrogen compound metabolic process
GO:0018130	6.2E-07	heterocycle biosynthetic process
GO:1901362	7.3E-07	organic cyclic compound biosynthetic process
GO:0019438	1.1E-06	aromatic compound biosynthetic process
GO:0044271	3.6E-06	cellular nitrogen compound biosynthetic process
GO:0010468	7.8E-06	regulation of gene expression
GO:0006270	2.3E-05	DNA replication initiation
GO:0008150	2.4E-05	biological_process
GO:0016782	6.3E-05	transferase activity, transferring sulfur-containing groups
GO:1901363	2.3E-04	heterocyclic compound binding
GO:0097159	2.3E-04	organic cyclic compound binding
GO:0003824	5.4E-04	catalytic activity
GO:0016020	7.3E-04	membrane
GO:0016779	8.5E-04	nucleotidyltransferase activity
GO:0034645	1.2E-03	cellular macromolecule biosynthetic process

GO:0005971	1.3E-03	ribonucleoside-diphosphate reductase complex
GO:0005694	2.3E-03	chromosome
GO:0043168	2.3E-03	anion binding
GO:0006457	2.6E-03	protein folding
GO:0046983	4.1E-03	protein dimerization activity
GO:0009059	4.5E-03	macromolecule biosynthetic process
GO:0016070	4.8E-03	RNA metabolic process
GO:0018106	5.2E-03	peptidyl-histidine phosphorylation
GO:0004673	5.2E-03	protein histidine kinase activity
GO:0044249	5.7E-03	cellular biosynthetic process
GO:0016757	6.1E-03	transferase activity, transferring glycosyl groups
GO:0016817	6.3E-03	hydrolase activity, acting on acid anhydrides
GO:0048037	6.6E-03	cofactor binding
GO:0005215	6.6E-03	transporter activity
GO:0044710	6.6E-03	single-organism metabolic process
GO:0008094	7.1E-03	DNA-dependent ATPase activity
GO:0016787	1.5E-02	hydrolase activity
GO:0018202	1.9E-02	peptidyl-histidine modification

Upregulated

ID	p.adjusted	Biological function
GO:0005840	6.0E-19	ribosome
GO:0030529	7.1E-15	ribonucleoprotein complex
GO:0006414	1.1E-13	translational elongation
GO:0005737	1.3E-11	cytoplasm
GO:0022884	2.6E-10	macromolecule transmembrane transporter activity
GO:0005198	6.9E-10	structural molecule activity
GO:0006412	2.2E-09	translation
GO:0044444	2.9E-09	cytoplasmic part
GO:0071806	4.9E-08	protein transmembrane transport
GO:0008233	4.9E-08	peptidase activity
GO:0019538	9.8E-08	protein metabolic process
GO:0008320	1.2E-07	protein transmembrane transporter activity
GO:0015450	1.6E-07	PP-bond-hydrolysis-driven protein transmembrane transporter activity
GO:0043228	4.1E-06	non-membrane-bounded organelle
GO:0043232	9.3E-06	intracellular non-membrane-bounded organelle
GO:0009306	2.3E-04	protein secretion
GO:0045454	2.3E-04	cell redox homeostasis
GO:0003735	2.3E-04	structural constituent of ribosome
GO:0019725	3.9E-03	cellular homeostasis
GO:0008565	6.4E-03	protein transporter activity
GO:0046700	1.5E-02	heterocycle catabolic process
GO:0044106	1.5E-02	cellular amine metabolic process
GO:0004674	1.7E-02	protein serine/threonine kinase activity
GO:0051082	1.7E-02	unfolded protein binding
GO:0018212	1.7E-02	peptidyl-tyrosine modification
GO:1901361	1.7E-02	organic cyclic compound catabolic process

A.31-B.6

Downregulated

ID	p.adjusted	Biological function
GO:0006414	2.2E-16	translational elongation
GO:0006412	1.7E-10	translation
GO:0005737	3.8E-10	cytoplasm
GO:0030529	7.3E-10	ribonucleoprotein complex

GO:0044444	7.4E-10	cytoplasmic part
GO:0005840	1.2E-09	ribosome
GO:0005198	1.5E-09	structural molecule activity
GO:0043228	1.7E-07	non-membrane-bounded organelle
GO:0008233	1.8E-07	peptidase activity
GO:0043232	2.8E-07	intracellular non-membrane-bounded organelle
GO:0022884	7.8E-07	macromolecule transmembrane transporter activity
GO:0071806	3.2E-05	protein transmembrane transport
GO:0015450	5.0E-05	PP-bond-hydrolysis-driven protein transmembrane transporter activity
GO:0008320	1.5E-04	protein transmembrane transporter activity
GO:0019205	4.0E-04	nucleobase-containing compound kinase activity
GO:0009309	4.0E-04	amine biosynthetic process
GO:0046939	5.2E-04	nucleotide phosphorylation
GO:0044281	5.2E-04	small molecule metabolic process
GO:0044106	1.3E-03	cellular amine metabolic process
GO:0009306	1.3E-03	protein secretion
GO:0043492	2.9E-03	ATPase activity, coupled to movement of substances
GO:0051082	5.9E-03	unfolded protein binding
GO:0016776	7.4E-03	phosphotransferase activity, phosphate group as acceptor
GO:0004674	7.6E-03	protein serine/threonine kinase activity
GO:0016301	1.2E-02	kinase activity
GO:0046906	1.3E-02	tetrapyrrole binding
GO:0019899	1.4E-02	enzyme binding
GO:0004690	1.6E-02	cyclic nucleotide-dependent protein kinase activity
GO:0072527	1.6E-02	pyrimidine-containing compound metabolic process
GO:0008565	1.6E-02	protein transporter activity
GO:0055086	1.6E-02	nucleobase-containing small molecule metabolic process
GO:0072528	1.9E-02	pyrimidine-containing compound biosynthetic process
GO:0032991	1.9E-02	macromolecular complex

Upregulated

ID	p.adjusted	Biological function
GO:0006260	1.09E-13	DNA replication
GO:0071897	1.09E-13	DNA biosynthetic process
GO:0006259	1.31E-11	DNA metabolic process
GO:0090304	3.58E-11	nucleic acid metabolic process
GO:0006725	1.25E-10	cellular aromatic compound metabolic process
GO:1901360	2.08E-09	organic cyclic compound metabolic process
GO:0016817	2.38E-09	hydrolase activity, acting on acid anhydrides
GO:0034641	3.08E-08	cellular nitrogen compound metabolic process
GO:0046483	4.90E-08	heterocycle metabolic process
GO:0016491	1.11E-07	oxidoreductase activity
GO:0043231	1.29E-06	intracellular membrane-bounded organelle
GO:0043227	1.56E-06	membrane-bounded organelle
GO:0006139	1.56E-06	nucleobase-containing compound metabolic process
GO:0008150	2.17E-06	biological_process
GO:0048037	5.50E-06	cofactor binding
GO:0016779	6.78E-06	nucleotidyltransferase activity
GO:0043168	2.07E-05	anion binding
GO:0016787	2.08E-05	hydrolase activity
GO:0016054	2.13E-05	organic acid catabolic process
GO:0044282	2.42E-05	small molecule catabolic process
GO:0006082	3.29E-05	organic acid metabolic process
GO:0006629	4.39E-05	lipid metabolic process
GO:0015299	4.63E-05	solute:proton antiporter activity
GO:0006807	5.71E-05	nitrogen compound metabolic process

GO:0050660	1.86E-04	flavin adenine dinucleotide binding
GO:0055114	1.91E-04	oxidation-reduction process
GO:0016887	2.13E-04	ATPase activity
GO:0043413	2.69E-04	macromolecule glycosylation
GO:0006520	2.69E-04	cellular amino acid metabolic process
GO:0046395	3.00E-04	carboxylic acid catabolic process
GO:0044710	3.00E-04	single-organism metabolic process
GO:0003676	3.28E-04	nucleic acid binding
GO:0042579	3.31E-04	microbody
GO:0016769	3.31E-04	transferase activity, transferring nitrogenous groups
GO:0034440	4.25E-04	lipid oxidation
GO:0016042	4.79E-04	lipid catabolic process
GO:0044242	5.50E-04	cellular lipid catabolic process
GO:0009059	6.04E-04	macromolecule biosynthetic process
GO:0034645	7.19E-04	cellular macromolecule biosynthetic process
GO:0022613	7.65E-04	ribonucleoprotein complex biogenesis
GO:0016070	8.56E-04	RNA metabolic process
GO:0044255	9.29E-04	cellular lipid metabolic process
GO:0004386	1.03E-03	helicase activity
GO:0046834	1.69E-03	lipid phosphorylation
GO:0009100	1.84E-03	glycoprotein metabolic process
GO:0006486	2.36E-03	protein glycosylation
GO:0005575	2.86E-03	cellular_component
GO:0003824	3.06E-03	catalytic activity
GO:0019395	3.51E-03	fatty acid oxidation
GO:0072329	3.85E-03	monocarboxylic acid catabolic process
GO:0030684	3.98E-03	preribosome
GO:0016627	4.20E-03	oxidoreductase activity, acting on the CH-CH group of donors
GO:0022804	4.54E-03	active transmembrane transporter activity
GO:0015298	4.55E-03	solute:cation antiporter activity
GO:0008237	4.85E-03	metallopeptidase activity
GO:0008289	4.85E-03	lipid binding
GO:0030170	4.85E-03	pyridoxal phosphate binding
GO:0016757	4.85E-03	transferase activity, transferring glycosyl groups
GO:0006270	5.03E-03	DNA replication initiation
GO:0015662	5.20E-03	ATPase activity, coupled to transmembrane movement of ions
GO:0051056	6.57E-03	regulation of small GTPase mediated signal transduction
GO:0044249	6.98E-03	cellular biosynthetic process
GO:1901363	7.65E-03	heterocyclic compound binding
GO:0097159	7.65E-03	organic cyclic compound binding
GO:0006644	7.72E-03	phospholipid metabolic process
GO:0009101	7.95E-03	glycoprotein biosynthetic process
GO:0009062	8.17E-03	fatty acid catabolic process
GO:0005215	8.21E-03	transporter activity
GO:0016741	8.78E-03	transferase activity, transferring one-carbon groups
GO:1902531	8.78E-03	regulation of intracellular signal transduction
GO:0005730	8.86E-03	nucleolus
GO:0009966	9.14E-03	regulation of signal transduction
GO:0005543	9.60E-03	phospholipid binding

A.31-H.14

Downregulated

**ID**	**p.adjusted**	**Biological function**
GO:0022884	1.3E-08	macromolecule transmembrane transporter activity
GO:0071806	2.3E-08	protein transmembrane transport

GO:0015450	4.8E-07	PP-bond-hydrolysis-driven protein transmembrane transporter activity
GO:0016020	1.6E-05	membrane
GO:0009306	2.9E-05	protein secretion
GO:0008565	2.9E-05	protein transporter activity
GO:0008320	2.9E-05	protein transmembrane transporter activity
GO:0008233	5.0E-03	peptidase activity
GO:0032940	1.4E-02	secretion by cell
GO:0070001	1.4E-02	aspartic-type peptidase activity
GO:0022804	1.8E-02	active transmembrane transporter activity

Upregulated

**ID**	**p.adjusted**	**Biological function**
GO:0006508	1.8E-04	proteolysis
GO:0003824	1.8E-04	catalytic activity
GO:0016491	7.1E-04	oxidoreductase activity
GO:0071897	1.4E-03	DNA biosynthetic process
GO:0006260	1.5E-03	DNA replication
GO:0043227	3.3E-03	membrane-bounded organelle
GO:0043231	3.3E-03	intracellular membrane-bounded organelle
GO:0048037	3.5E-03	cofactor binding
GO:0006082	6.5E-03	organic acid metabolic process
GO:0044710	1.1E-02	single-organism metabolic process
GO:0016769	1.1E-02	transferase activity, transferring nitrogenous groups
GO:1901575	1.4E-02	organic substance catabolic process
GO:0030170	1.4E-02	pyridoxal phosphate binding
GO:0008150	1.4E-02	biological_process
GO:0016817	1.4E-02	hydrolase activity, acting on acid anhydrides
GO:0044699	1.5E-02	single-organism process
GO:0005975	1.5E-02	carbohydrate metabolic process
GO:0006259	1.5E-02	DNA metabolic process
GO:0055114	1.8E-02	oxidation-reduction process
